# Multiproxy bioarchaeological analyses reveal subsistence shift in Bronze Age Central Europe

**DOI:** 10.1126/sciadv.aeh1079

**Published:** 2026-09-09

**Authors:** Margaux L. C. Depaermentier, Przemysław Makarowicz, Anita Szczepanek, Christophe Snoeck, Marine Morvan, Zdzislaw Belka, Michael Kempf, Łukasz Pospieszny, Halina Taras, Maciej Chyleński, Jacek Górski, Tomasz Goslar, Ryszard Grygiel, Paweł Jarosz, Anna Juras, Anna Lasota-Kuś, Andrzej Matoga, Marek Nowak, Marcin M. Przybyła, Krzysztof Tunia, Piotr Włodarczak, Jarosław Wilczyński, Irena Wójcik, Giedrė Motuzaitė Matuzevičiūtė

**Affiliations:** ^1^ Vilnius University, Faculty of History, Department of Archaeology, Universiteto g. 7, LT-01513 Vilnius, Lithuania.; ^2^ Faculty of Archaeology, Adam Mickiewicz University in Poznań, Uniwersytetu Poznańskiego 7, 61-614 Poznań, Poland.; ^3^ Institute of Archaeology and Ethnology, Polish Academy of Sciences, Sławkowska 17, 31-016 Kraków, Poland.; ^4^ Archaeology, Environmental Changes and Geo-Chemistry, Vrije Universiteit Brussel, Pleinlaan 2, Brussels 1050, Belgium.; ^5^ Isotope Research Unit, Adam Mickiewicz University, Krygowskiego 10, 61-680 Poznań, Poland.; ^6^ Department of Geography, University of Cambridge, Cambridge, UK.; ^7^ Institute of Archaeology, University of Gdańsk, ul. Bielańska 5, 80-851 Gdańsk, Poland.; ^8^ School of Chemistry, University of Bristol, Cantock’s Close, Bristol BS8 1TS, UK.; ^9^ Institute of Archaeology, Maria Curie–Skłodowska University, M. Curie-Skłodowska Sq. 4, 20-031 Lublin, Poland.; ^10^ Institute of Human Biology and Evolution, Faculty of Biology, Adam Mickiewicz University in Poznań, Uniwersytetu Poznańskiego 6, 61-614 Poznań, Poland.; ^11^ Archaeological Museum in Cracow, Senacka 3, 31-002 Kraków, Poland.; ^12^ Poznań Radiocarbon Laboratory, Poznań Park of Science and Technology, Rubież 46, 61-612 Poznań, Poland.; ^13^ Faculty of Geographical and Geological Sciences, Adam Mickiewicz University in Poznań, Krygowskiego 10, 61-680 Poznań, Poland.; ^14^ Museum of Archaeology and Ethnography in Łódź, Wolności Sq. 14, 91-415 Łódź, Poland.; ^15^ Institute of Archaeology, Jagiellonian University, 11 Gołębia St., 31-007 Kraków, Poland.; ^16^ Dolmen S.C., 8/3 Serkowskiego Sq., 30-512 Kraków, Poland.; ^17^ Institute of Systematics and Evolution of Animals, Polish Academy of Sciences, Sławkowska 17, 31-016 Kraków, Poland.

## Abstract

Changes in past subsistence strategies reflect the adaptation of human societies to climatic, environmental, and cultural settings. Despite being a resistant, flexible, and nutritive crop, millet only sporadically became a staple crop in Europe and is nearly forgotten today. This raises the question of the cultural dimensions associated with the adoption or rejection of millet. We use a multi-isotope dataset from Bronze Age Poland (2200 to 1000 BCE) coupled with radiocarbon dates to identify dietary practices and associate them with cultural and environmental conditions. We (i) revise the earliest evidence for millet consumption in Europe to 1590 ± 50 calibrated years (cal) BCE instead of 1460 ± 40 cal BCE, (ii) reconstruct dietary shifts at the group and individual level, (iii) suggest environmental instead of cultural mechanisms behind millet adoption, and (iv) reveal land-use shifts associated with millet cultivation, showing no strong evidence for a demic diffusion model. The gained insights into crop integration processes can help in discussing coping strategies for present-day challenges.

## INTRODUCTION

Subsistence strategies are shaped by the incorporation of diverse staple crops; however, little is known about how new plants were integrated into food production systems of past populations. Taking the example of millets (*Setaria italica* and *Panicum miliaceum*), these drought-resistant and fast-growing summer crops, domesticated in Northern China around 8000 years ago (*1*, *2*), had reached Europe by around 1550 BCE (*3*–*5*), after spreading across Eurasia (*4*, *6*, *7*). More precisely, millet spread through the Inner Asian Mountain Corridor during the third millennium BCE, dispersing along river valleys and fertile alluvial fans of the Tien Shan, Pamir, and Altai Mountains (*8*–*11*), and ultimately reached Europe via the Caspian Steppe and present-day Ukraine (*12*–*14*). Millet dispersal across Central Asia has been associated with mobile pastoral economies (*15*), yet its introduction in Europe is also related to farmer communities (*14*). Despite its ecological flexibility and high nutritious status, broomcorn millet (*P. miliaceum*; hereafter “millet”) became only sporadically a staple crop in Europe over the past (*16*–*21*) and is now absent from most present-day dietary and agricultural systems. The earliest evidence for a substantial dietary intake of millet in Europe has been identified by means of carbon isotope analyses in southern Poland at around 1460 ± 40 calibrated years (cal) BCE (*16*). Although a major part of the regional population adopted this crop, some communities did not consume it at all, a phenomenon that was observed in other regions as well (*4*, *22*–*24*). This raises the question of the still underinvestigated cultural dimensions associated with the adoption or rejection of millet.

**Fig. 1.**
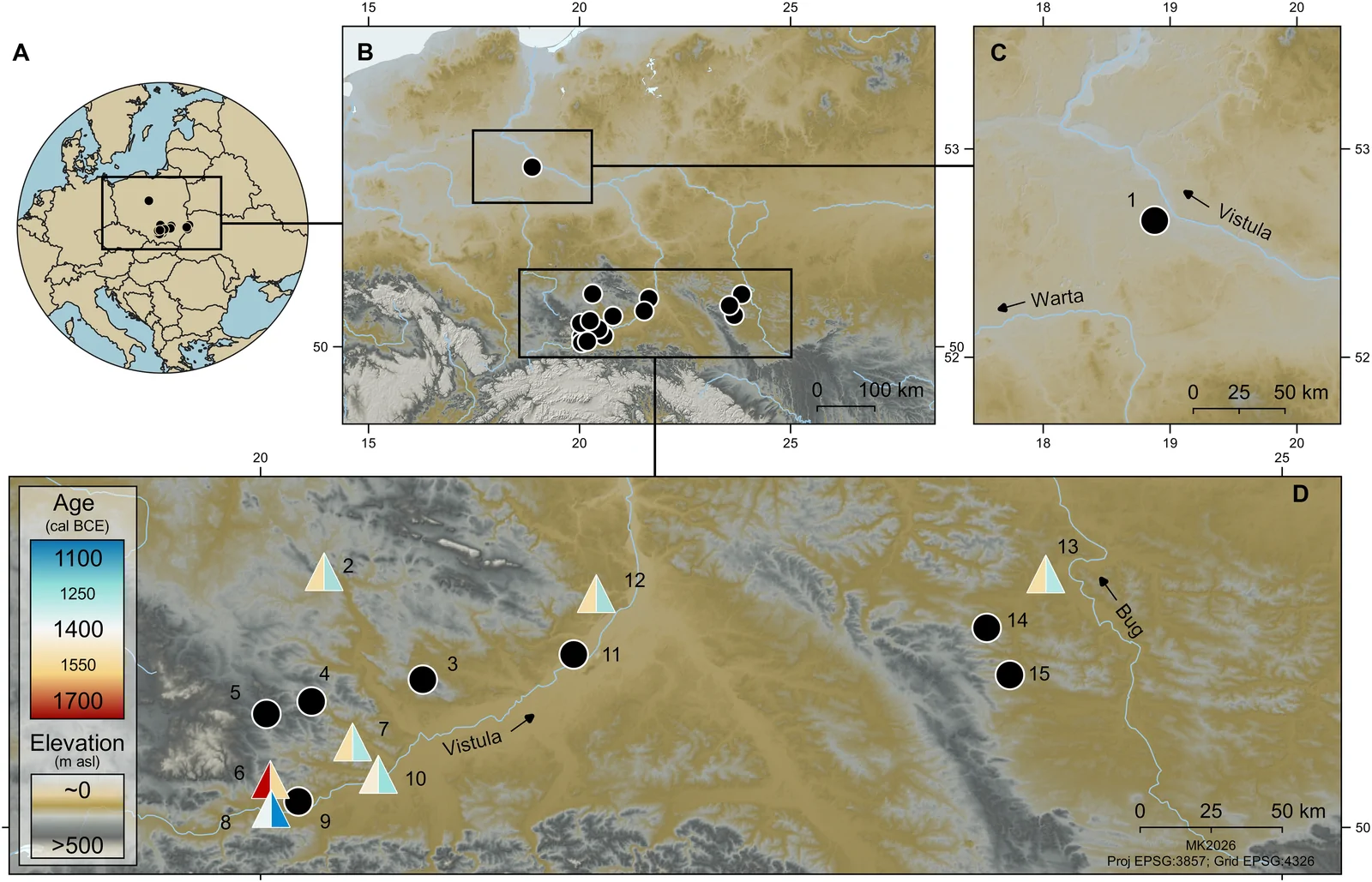
Site location within the study area with topography and major hydrological conditions. (**A**) Location within Europe and the boundaries of modern countries. (**B**) Site distribution in Central Europe with topography and main streams. (**C**) Northern part of the study area. (**D**) Southern part of the study area. The numbers refer to the site names as follow: 1, Gustorzyn; 2, Bocheniec; 3, Żerniki Górne; 4, Pieczeniegi/Rzemiędzice; 5, Miechów; 6, Pielgrzymowice; 7, Słonowice; 8, Kraków Nowa Huta (KNH) Mogiła; 9, Kraków Nowa Huta-Cło; 10, Koszyce; 11, Świniary Stare; 12, Dacharzów; 13, Brodzica; 14, Zubowice; 15, Podlodów. The black dots on (C) and (D) represent sites with no isotopic evidence for C_4_ diet. The triangles on (D) represent sites with isotopic evidence for C_4_ consumption. The triangle’s color represents the earliest date of millet consumption at each site with the left half showing the minimum age cal BCE and the right half the maximum age cal BCE. The exact dates are summarized in Table 1. This is an original figure created by M.K. in QGIS, using a resampled 500-m digital elevation model (in meters above sea level: m asl) from the Global Multi-Resolution Terrain Elevation Data 2010 (GMTED2010, http://doi.org/10.5066/F7J38R2N, last accessed 29 May 2026). Rivers and lakes layers are made with Natural Earth, using free vector and raster map data from naturalearthdata.com, last accessed 29 May 2026.

To address these questions, we applied an interdisciplinary and multiproxy approach to comparing the pioneer millet-eaters with contemporaneous non–millet-eaters from Poland (Fig. 1). We first traced millet consumption down to the individual level using carbon isotope analyses. Because of its C_4_ photosynthetic pathway, millet has a considerably more elevated carbon isotope ratio [expressed as δ^13^C in per mil (‰) versus Vienna Pee Dee belemnite (VPDB), ranging from −14.0 to −10.0‰] than C_3_ plants such as wheat and barley (with δ^13^C values from −35 to −23‰) (*25*, *26*). Because the isotopic composition of food gets transferred to the body tissues of the consumer according to known fractionation processes (*27*, *28*), a C_4_ input into a C_3_ diet can be isotopically identified from bone or dentin collagen δ^13^C values above −18.0‰ (*29*, *30*) and from enamel carbonate with δ^13^C values above −10.0‰ (*31*, *32*). With the combined nitrogen isotope data (δ^15^N), we can rule out other factors such as marine food (*27*). Because they reflect the environmental conditions in which the food ingested during childhood was produced, we further used strontium (^87^Sr/^86^Sr) and oxygen (δ^18^O) isotope data from human dental enamel to track the geographical origin of the consumed food (*33*). In particular, ^87^Sr/^86^Sr values are primarily determined by geological, pedological, and hydrological conditions associated with the provenience of the food ingested during childhood (*33*–*35*), while oxygen reflects more directly the diverse water sources and regional climatic conditions (*36*, *37*). Coupled with published genetic data (*16*, *38*, *39*) and compared to the environmental background, this informs on human mobility patterns and land-use practices. Information about these people’s cultural and biological backgrounds were collected from newly generated paleoproteomic (i.e., amelogenin protein) and existing genetic (*16*, *38*, *39*), archaeological, and anthropological data (*40*–*51*). This multiproxy and multitissue approach is a golden opportunity to obtain multifaceted insights into the cultural and ecological conditions related to the initial millet consumption in Poland, while filling gaps in bioarchaeological records for the Middle Bronze Age in Central Europe.

## RESULTS

The results of the multiproxy isotope analyses, radiocarbon dating, and amelogenin protein analyses are available from data S1 [see also (*52*)]. For each individual and archaeological site, we integrated previously published stable, radiogenic, and radiocarbon isotope data (*13*, *16*, *40*, *53*–*55*) as well as existing archaeological and anthropological data (*40*–*51*). For 78 individuals, existing genetic information was included as well (*16*, *38*, *39*). Table 1 summarizes the main outcome by site. The identification of dietary habits is primarily based on the results of the multitissue δ^13^C values, as extensively defined in Materials and Methods.

**Table 1.** Overview of the main outcomes by site. The table presents the newly produced data complemented by previously published data from the same sites. The sites with isotopic evidence for C_4_ inputs are marked by triangles on Fig. 1D. The sites with no clear isotopic evidence for C_4_ inputs are marked by black dots on Fig. 1 (C and D). The detailed results by individual can be found in data S1.

Diet at site	Site name	Number on Fig. 1	Chronological span (years BCE)	Earliest ^14^C-date for millet input		Number of investigated individuals (combining new and published datasets)			Individuals on a C_3_ diet		Individuals changing from a C_3_ diet to C_4_ inputs		Individuals with C_4_ input		Comment
				Mean date ± SD (years B.P.)	Min. to max. age (years cal BCE)	In total	Among total: with δ^13^C values	Among total: with ^87^Sr/^86^Sr and/or δ^18^O values	Count	% at the site	Count	% at the site	Count	% at the site	
Isotopic evidence for C_4_ inputs	Bocheniec 2	2	1500–1100	3135 ± 30	1497–1302	15	14	8	0	0	1	7%	13	93%	
	Brodzica 19	13	1500–1300	3145 ± 30	1499–1311	3	3	2	0	0	0	0	3	100%	Percentages not relevant/reliable: small sample size.
	Dacharzów 1	12	1500–1300	3135 ± 35	1499–1299	9	9	6	3	33%	1	11%	5	56%	
	Koszyce 3	10	1450–1100	3100 ± 35	1434–1278	7	7	6	1	14%	0	0	6	86%	Percentages not relevant/reliable: small sample size.
	Kraków Nowa Huta Mogiła 55	8	1500–1100	2995 ± 35	1385–1116	2	2	0	0	0	0	0	2	100%	Percentages not relevant/reliable: small sample size.
	Pielgrzymowice 9	6	1800–1600	NA	NA	1	1	1	1	100%	0	0	0	0	Predating millet arrival in Poland. Percentages not relevant/reliable: small sample size.
			1700–1300	3320 ± 35	1687–1507	13	13	12	1	7%	3	23%	9	69%	
	Słonowice 5	7	1800–1600	NA	NA	1	1	1	1	100%	0	0	0	0	Predating millet arrival in Poland. Percentages not relevant/reliable: small sample size.
			1700–1300	3128 ± 30	1496–1297	9	9	9	6	66%	1	11%	2	22%	The individual who changed from a C_3_ diet in childhood to C_4_ inputs in adulthood remains just below the threshold for C_4_ identification: This could be a small C_4_ input or a dietary change only shortly predating death.
No isotopic evidence for C_4_ inputs	Kraków Nowa Huta-Cło 65	9	1750–1500	NA	NA	3	3	2	3	100%	0	0	0	0	Percentages not relevant/reliable: small sample size.
	Miechów 3	5	1700–1200	NA	NA	16	16	13	16	94%	0	0	0	0	
			1400–400	NA	NA	3	3	0	2				1		Percentages not relevant/reliable: small sample size. Potential C_4_ signal close to threshold.
	Gustorzyn 1	1	1700–1200	NA	NA	17	12	17	12	100%	0	0	0	0	The site is located further north in Poland.
	Pieczeniegi/Rzemiędzice	4	2100–1700	NA	NA	1	1	1	1	100%	0	0	0	0	Predating millet arrival in Poland. Percentages not relevant/reliable: small sample size.
	Podlodów 2	15	1850–1600	NA	NA	3	3	0	3	100%	0	0	0	0	Percentages not relevant/reliable: small sample size.
	Świniary Stare 1	11	2200–1600	NA	NA	15	14	10	14	100%	0	0	0	0	Predating millet arrival in Poland.
	Żerniki Górne 1	3	2600–2200	NA	NA	8	0	8	NA	NA	NA	NA	NA	NA	Predating millet arrival in Poland. No dietary information.
			2200–1800	NA	NA	16	16	10	16	100%	0	0	0	0	Predating millet arrival in Poland.
			1700–1100	NA	NA	39	38	20	38	100%	0	0	0	0	Includes one Late Bronze Age individual (1400–1100 BCE).
	Zubowice 1A	14	2300–1800	NA	NA	4	4	1	4	100%	0	0	0	0	Predating millet arrival in Poland. Percentages not relevant/reliable: small sample size.
			1500–1300	NA	NA	7	2	6	2	100%	0	0	0	0	Predating millet arrival in Poland. Percentages not relevant/reliable: small sample size.
Sum						Individual in total	Individual with δ^13^C values	Individual with ^87^Sr/^86^Sr and/or δ^18^O values	Individuals on a C_3_ diet		Individuals changing from a C_3_ diet to C_4_ inputs		Individuals with C_4_ input		Comment
Sum	All sites		2300–1100			192	171	133	124	73%	6	4%	41	24%	Including Eneolithic and Late Bronze Age/Iron Age individuals
	Early Bronze Age		2300–1600			41	40	24	40	100%					Predating millet arrival in Poland.
	Middle Bronze Age		1700–1200	3320 ± 35	1687–1507	140	128	101	82	64%	6	5%	40	31%	Includes the Late Bronze Age individual from Żerniki Górne 1 (1400–1100 BCE).

### Carbon and nitrogen isotope analyses

In this section, we first present the results of the newly generated carbon (δ^13^C versus VPDB) and nitrogen (δ^15^N versus AIR) isotope analyses, which encompass a total of 139 dentin collagen samples, 52 bone collagen samples, and 56 enamel samples, deriving from a total of 138 individuals. The valid results from the newly generated dataset are completed by 99 published collagen isotope data from the same sites (*16*, *40*, *53*–*55*). Among the 99 human individuals providing preexisting stable isotope datasets, these are 66 individuals for which new data were additionally generated in this study and 33 individuals for which no additional data were produced. This results in a total of 171 individuals with valid dietary information. Detailed results for both newly generated and previously published data are listed in data S1 [see also (*52*)], along with the related references.

Among the valid samples analyzed in this study, the dentin (*n* = 122 samples from 121 individuals) δ^15^N values span from 7.5 to 12.9‰ (average, 10.4 ± 1.2‰), and the bone (*n* = 52) collagen δ^15^N values span from 7.4 to 11.5‰ (average, 9.7 ± 1.0‰) (data S1). The dentin δ^13^C values range from −21.6 to −13.7‰ (average, −19.3 ± 1.8‰), and the bone collagen δ^13^C values range from −20.8 to −14.4‰ (average, −17.7 ± 2.2‰). While the δ^13^C values are used for identifying C_4_ plant consumption (see below), the paired δ^15^N and δ^13^C results enable us to rule out the impact of marine food or aridity on the associated δ^13^C values. Except for six teeth, the dentin samples showing δ^15^N values of 11.0‰ or above (*n* = 37) derive from first molars and therefore likely reflect a breastfeeding signal (*36*). The same applies for the bone of the infant individual that shows the most elevated δ^15^N value among bone collagen samples (11.5‰). The other six individuals showing elevated dentin δ^15^N values are either Early Bronze Age individuals showing no elevated δ^13^C values or outlier individuals (see below and data S1).

When completing the newly generated data with existing datasets from the same investigated Bronze Age sites (*16*, *40*, *53*–*55*), we could compare the carbon isotope values of the different tissues, which represent different life stages: The petrous bone represent the in utero life, the dentin and enamel samples represent different stages of the childhood, and the bone samples represent the last years (up to decades) of life (*56*, *57*). In total, 66 individuals present both dentin and bone collagen δ^13^C data. The difference δ^13^C_bone_-δ^13^C_dentin_ overall ranges from −3.2‰ (MIL1_230) to 5.6‰ (MIL1_207) and averages at 0.4 ± 1.3‰. This shows that for 65% of these individuals (*n* = 66), the collagen δ^13^C values increased between childhood and the last years of life, often reflecting an appearance or increase in C_4_ intake over life (data S1 and fig. S1).

In addition to the dietary information from the collagen samples, which derives from the protein part of the diet, the δ^13^C values from the enamel reflect the whole part of the diet and therefore provide complementary insights into the role C_3_ versus C_4_ plants in the diet (*28*). The enamel carbonate δ^13^C values span widely from −14.7 to −6.4‰ and average at −10.9 ± 2.6‰. By comparing the collagen and enamel δ^13^C values, it was possible to reinterpret some elevated δ^13^C values from petrous bone collagen (e.g., MIL1_335; see Materials and Methods and fig. S2), to identify indirect or particularly low degrees of C_4_ inputs, and dietary changes over people’s lifetime (see figs. S1 and S2 and related explanations in Materials and Methods).

**Fig. 2.**
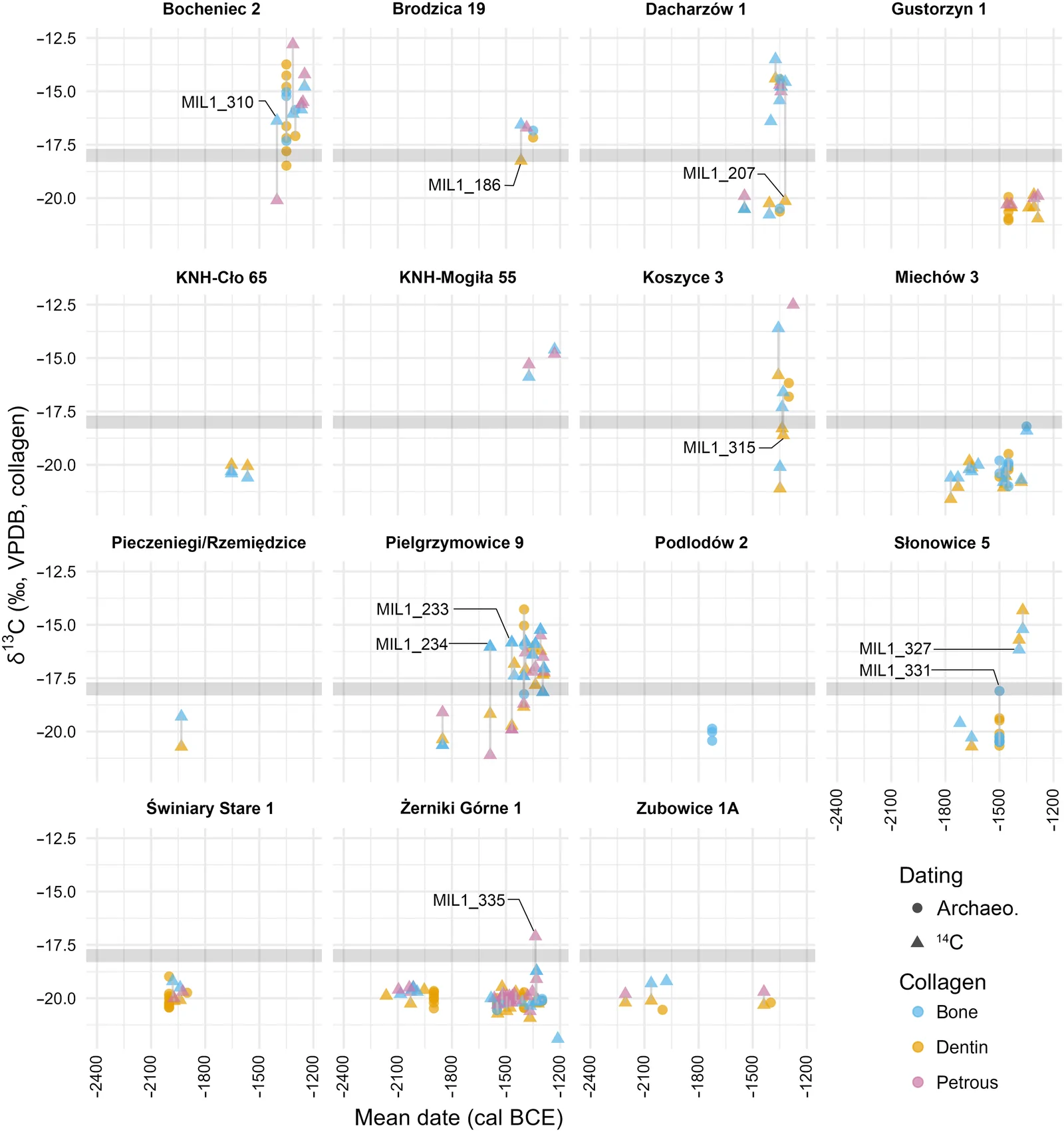
Comparison between collagen δ^13^C values and the mean absolute date (BCE) of the individual at each site, using the whole dataset. The samples belonging to the same individual are connected with a gray line. The shape of the point informs about the radiocarbon (triangle) versus archaeological (dots) dating method. The span for radiocarbon dates is mostly between 100 and 200 years (but up to 300 years), while the range for archaeological dates spans from 100 to 600 years. The detailed results can be found in data S1. The color of the point informs about the body part selected for collagen stable isotope analysis, which reflects different life stages: the bone samples representing the last years or decades of life, the dentin samples representing different stages of the childhood, and the petrous bone representing the in utero life (*56*, *57*). The gray horizontal shaded areas mark the δ^13^C values at around −18.0‰, a common threshold for identifying C_4_ dietary inputs in Poland (*30*). The individuals specifically discussed in the text are labeled.

Beyond the individual level, it was possible to distinguish (contemporaneous) sites at which millet consumers were isotopically identified from sites with exclusively people on a C_3_ diet (Table 1 and Fig. 1). Among sites with C_4_ consumption, it is furthermore possible to highlight sites with clear dietary clusters (Dacharzów and Słonowice—both including one individual who changed diet) versus sites with people showing more diversified diets within the community (Bocheniec, Koszyce, and Pielgrzymowice) (figs. S1 and S2). Except at Kraków Nowa Huta Mogiła (*n* = 2), all sites with carbon isotopic evidence of millet introduction exhibit at least one individual who changed from a C_3_ to a mixed C_3_-C_4_ diet over their lifetime (Fig. 2 and figs. S1 and S2).

When including new and previously published data from the investigated sites (*n* = 171 individuals) (*16*, *40*, *53*–*55*), there are a total of 39 individuals who were on a C_3_ diet throughout their lives (Table 1 and data S1). Another 72 individuals show a C_3_ diet in their childhood but have no information about adulthood diet, and 12 more individuals have a C_3_ diet in adulthood, without information about childhood diet. Six individuals clearly changed from a C_3_ diet in childhood to C_4_ intakes during adulthood. In addition, 20 individuals had a diet with C_4_ inputs throughout their lives—among them, five individuals with only minor amounts. Ten individuals exhibit C_4_ inputs during their childhood and two during adulthood, but in each case without information about other life stages. The consumption of millet has considerably increased during the life of eight individuals and sensibly decreased over the lifetime of one single individual (MIL1_230). This makes a total of 124 individuals on a C_3_ diet, i.e., with no carbon isotopic evidence for C_4_ intake, and 47 individuals with minor to considerable C_4_ dietary inputs during at least one stage of their lives.

### Oxygen and strontium isotope analyses

The enamel δ^18^O values (versus VPDB) (*n* = 57 new samples from 56 individuals) range from −7.2 to −4.1‰ and average at −5.7 ± 0.6‰. The converted δ^18^O_DW_ (DW for drinking water) range from −11.3 to −6.2‰ (first quartile, −9.6‰; median, −8.8‰; mean, −8.9 ± 1.0‰; third quartile, −8.3‰) [data S1 and see also (*52*)]. Nearly all individuals therefore fall within the range typically found in this research area, i.e., between −11.4 and −7.0‰ (*54*, *58*). This includes the individuals with the lowest δ^18^O_DW_ values. The only outlier is MIL1_187, who exhibits a higher δ^18^O_DW_ value (−6.2‰) in the second molar (fig. S3). This value falls both outside the regionally expected isotopic range and outside the statistical distribution of the whole dataset (fig. S3), exceeding the usual variability expected within a group (*37*). This individual is an outlier in both enamel δ^18^O_DW_ and dentin collagen δ^15^N values and therefore possibly originated from a different region, despite the shared burial context with local and genetically related individuals (*38*) (data S1) and despite the seemingly local ^87^Sr/^86^Sr value measured in the same dental enamel, which is nearly identical to another individual from this grave. The equifinality of ^87^Sr/^86^Sr values across Europe can explain this situation (*33*, *35*, *59*), since the paired δ^18^O and δ^13^C values from the enamel are robust and reliable. The access to completely different water sources, a health issue, or radically different dietary habits that could explain such outlier δ^18^O value is therefore considered less likely than an outlier origin from a place with a similar strontium isotopic composition.

Among the 120 enamel samples coming from 107 individuals (including 13 samples that were measured in duplicates in both laboratories; see Materials and Methods) selected for strontium isotope analyses, only one single measurement failed at providing ^87^Sr/^86^Sr results (i.e., MIL1_176/MAK43). The dental enamel ^87^Sr/^86^Sr values of all other newly analyzed samples (*n* = 120 including duplicates) range from 0.7082 to 0.7129 and average at 0.7101 (1 SD = 0.0012) (data S1). The strontium concentration [Sr] (*n* = 43) varies widely from 29 to 280 parts per million (ppm) (mean, 105 ± 60 ppm).

We completed the dataset with the 35 already published ^87^Sr/^86^Sr values from individuals from the same sites (*38*, *54*), eight of whom were reanalyzed in this study to verify the interoperability of the datasets (see Materials and Methods), resulting in a total of 133 individuals. We then compared all individual Sr isotope ratios (*n* = 133 individuals) with the ^87^Sr/^86^Sr values of the local geologies (derived from environmental samples) and with the published ^87^Sr/^86^Sr values of other archaeological samples from the surroundings of each site as defined in Materials and Methods and identified 97 individuals matching the expected isotopic range around the site and 36 individuals with values falling outside their site’s range (fig. S4). Among these 97 individuals, one was an outlier in δ^18^O_DW_ (see MIL1_187 on figs. S3 and S4 and data S1).

### Radiocarbon dates combined with isotope data

This study builds on a large dataset of previously published and recalibrated radiocarbon dates (*n* = 105, including one that was redated in this study) (*16*, *38*, *40*, *53*–*55*), completed by 11 new radiocarbon dates. The results are available from data S1, along with the associated references for the published dates. In total, eight individuals integrated in this study belong to the Late Eneolithic Corded Ware Culture (∼2600 to 2200 BCE) and provided mobility data only. Thirty-nine individuals belong to the Early Bronze Age (∼2300 to 1700/1600 BCE), including 38 with dietary information and 22 with mobility information, i.e., 21 with both dietary and mobility information. Most individuals (*n* = 138) belong to the Middle Bronze Age (∼1700/1600 to 1200 BCE), with 126 providing dietary data, 100 providing mobility data, and, hence, 91 individuals with both mobility and dietary information. There are also three individuals belonging to the Early/Middle Bronze Age (with dietary and mobility information) and two individuals belonging to the Late Bronze Age (∼1200 to 800 BCE), with dietary information only. The two Iron Age individuals (800 to 400 BCE) from Miechów listed in data S1 are not integrated in the rest of the study due to their too recent chronology.

**Fig. 3.**
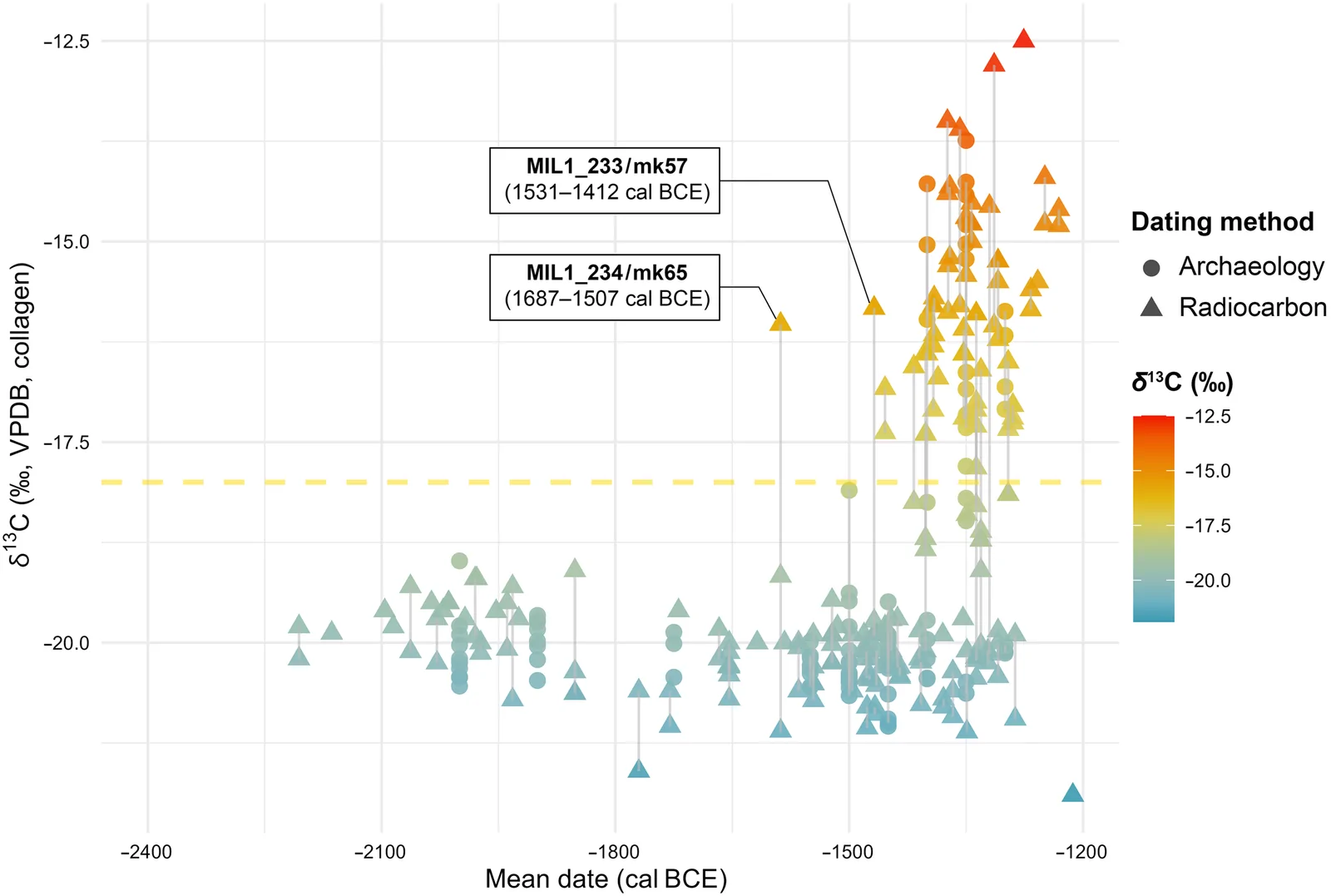
Chronological evolution of collagen δ^13^C values from Bronze Age Poland. The color reflects the variation in δ^13^C values. Dots are radiocarbon-dated individuals, and triangles are archaeologically dated individuals. The error for radiocarbon dates is mostly between 100 and 200 years (up to 300 years), and the archaeological dates have an uncertainty spanning from 100 to 600 years. The detailed results can be found in data S1. Collagen samples from different body parts of the same individual are connected by a gray line. The yellow dashed line marks the δ^13^C value at −18.0‰, a common threshold for identifying C_4_ dietary inputs in Poland (*30*). The two earliest ^14^C-dated millet-eaters are labeled.

By combining the radiocarbon data with the results of the multitissue isotope analyses (Fig. 2), it was possible to determine the earliest radiocarbon-dated evidence for isotopically visible millet consumption at each investigated site (Table 1 and Fig. 2). The earliest evidence for a substantial millet consumption in the whole region was found in the osteological remains of the female individual 2 from feature 748 at Pielgrzymowice 9 (see MIL1_234 on Figs. 2 and 3), who was originally identified as a C_3_-eater from the analysis of her petrous bone collagen (*16*), which reflects the in utero life. A C_3_-diet can, however, only be identified during her childhood, as confirmed from the new dentin collagen and dental enamel δ^13^C values of her first molar (data S1 and fig. S2), reflecting her diet until she was 10 years old at the latest (*57*). In contrast, her bone collagen δ^13^C value (−16.03‰) reveals that she integrated millet in her diet at a later stage of her life (Fig. 2). Since this woman died in the age of 40 to 50 years, it is not possible to determine when this dietary shift happened without samples from her second and third molar. However, her skeleton was complete, fully articulated, and clearly distant and distinguishable from the other burial in this grave. There is therefore no doubt that all samples belong to the same individual and that the isotopic differences among tissues reflect a dietary shift. This female individual was radiocarbon dated to 3320 ± 35 before the present (B.P.) [see Poz-104940 in (*8*)], i.e., 1588 ± 48 cal BCE. Another early date is found in the male individual in “feature 631” at the same site (MIL1_233 on Figs. 2 and 3), radiocarbon dated to 1468 ± 31 cal BCE [see Poz-104939 in (*16*)], and similarly shown a dietary shift with C_4_ inputs only during a later stage of life—i.e., after the complete formation of his first permanent molar and at the latest within the 5 years before death (Fig. 2). Again, this individual represents a single burial, and all samples thus belong without doubt to this one same individual.

**Fig. 4.**
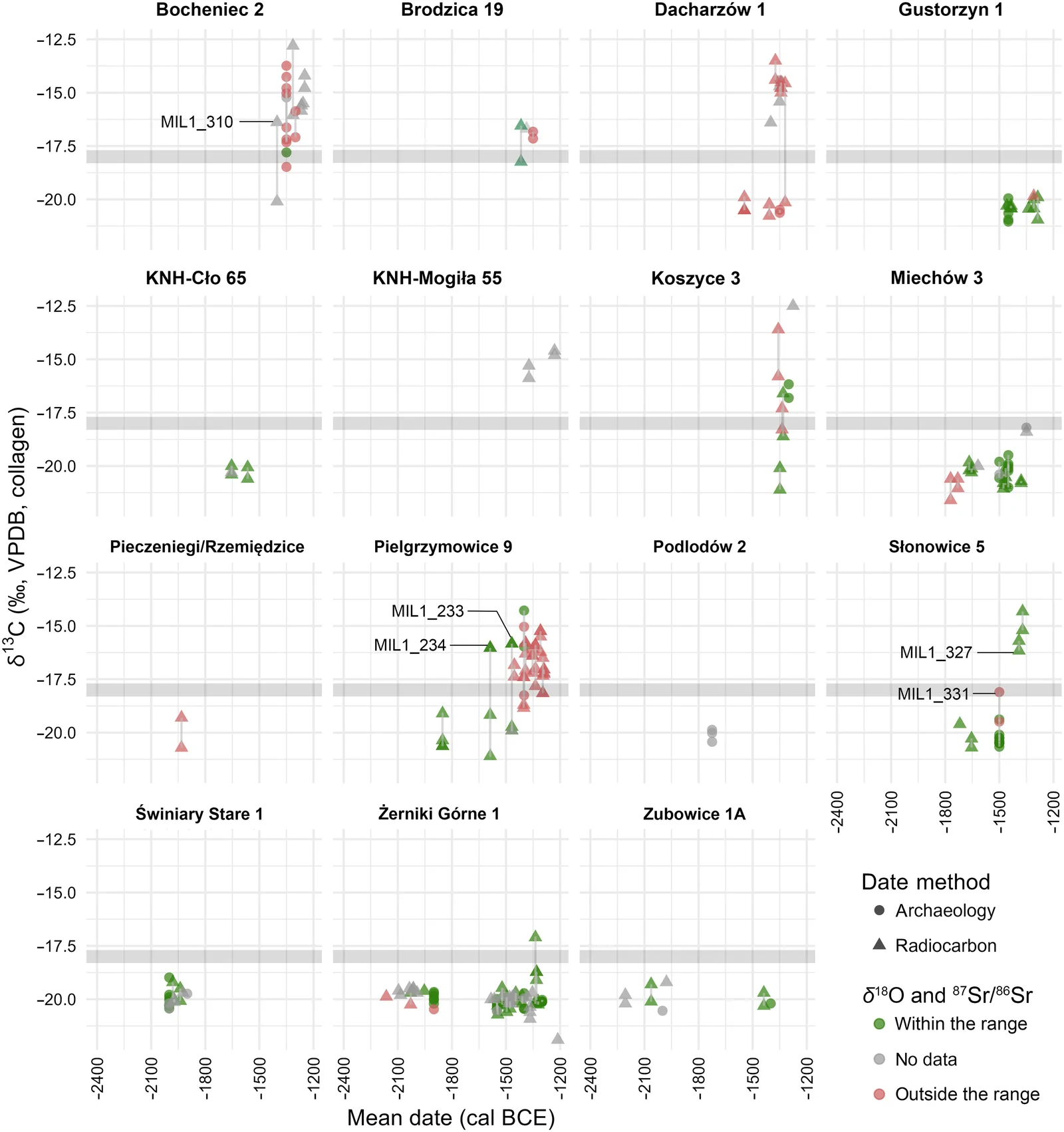
Chronological sequence of human collagen δ^13^C of individuals compared to the interpretation of their ^87^Sr/^86^Sr data at each site, using the whole dataset. The samples belonging to the same individual are connected with a light gray line. The shape of the point informs about the radiocarbon (triangles) versus archaeological (dots) dating method. The span for radiocarbon dates is mostly between 100 and 200 years (but up to 300 years), while the range for archaeological dates spans from 100 to 600 years. The detailed results can be found in data S1. The color of the signatures shows whether the ^87^Sr/^86^Sr ratio of each individual’s enamel falls within or outside the local range as defined for each site in Materials and Methods and depicted in fig. S4. The gray horizontal shaded areas mark the δ^13^C values at around −18.0‰, a common threshold for identifying C_4_ dietary inputs in Poland (*30*). Labeled individuals are discussed in the text.

The combined results of this multiproxy approach furthermore revealed that after 1500 BCE, nearly all individuals from sites with isotopic evidence for millet consumption had ^87^Sr/^86^Sr values falling outside the expected local range (as defined in Materials and Methods), except for the “local” C_4_ consumers at Słonowice (Fig. 4). The results of the kernel density estimation (KDE) models, a nonparametric statistical method used to estimate the underlying probability density function of a series of events and, hence, help identify cultural shifts over time (*60*), show that the radiocarbon-dated individuals on a diet including C_4_ inputs (without being originally on a C_3_ diet during their childhood) do not represent one single chronological event, as at least two individuals belong to an earlier phase and four individuals indicate a later chronological range (data S2A). The individuals associated with C_4_ consumption and with enamel ^87^Sr/^86^Sr values falling outside their site’s local Sr isotopic range also do not represent one single chronological event. This applies when considering the whole dataset at once (data S2B) or each site separately (data S2, C to E), except at Koszyce (data S2E), where the burials are largely contemporaneous. The radiocarbon-dated individuals from Bocheniec have no O or Sr isotope data (data S2F). The two radiocarbon-dated millet consumers from Brodzica were likely buried during the same event and are either nonlocal or without mobility data (Fig. 4 and data S2G). The two clear millet consumers from Słonowice are radiocarbon dated to a much later phase than the C_3_ community buried there before and are of local origin (Fig. 4 and data S2H).

### Combining multiple isotope data

**Fig. 5.**
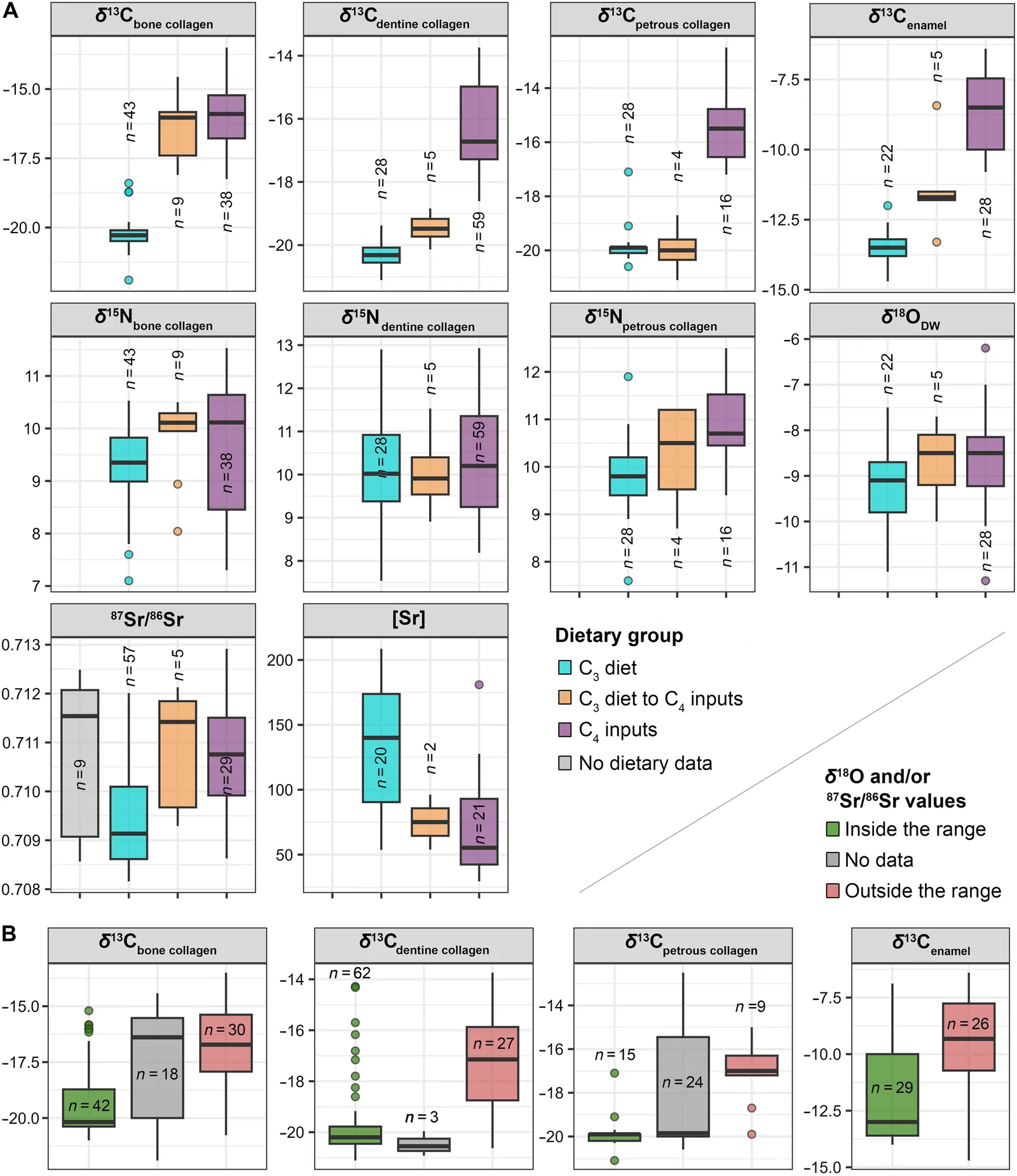
Isotopic variability between individuals with various diets and with various Sr isotopic backgrounds among samples dated to after 1700/1600 BCE. (**A**) Box plots showing the different isotopic distribution from each sample type of individuals with different dietary habits, i.e., individuals on a C_3_ diet, individuals changing from a C_3_ diet to C_4_ inputs, and individuals consuming C_4_ plants throughout their lives. Among individuals sampled for strontium isotope analyses, some had no dietary information because they were not sampled for stable carbon isotope analyses (see data S1 for details and Table 1 for a summary overview). (**B**) Box plots showing the different distribution in collagen and enamel δ^13^C values from the various tissues between individuals consuming food from different geographical origins as defined from the Sr and O isotopic composition of their dental enamel. Some individuals were sampled for collagen stable isotope analyses only and therefore do not provide such data (see data S1 for details and Table 1 for a summary overview). The various tissues reflect different life stages: The bone samples represent the last years or decades of life, the dentin and enamel samples represent different stages of the childhood, and the petrous bone represents the in utero life (*56*, *57*). The results of the respective statistical tests are available from Table 2.

The comparison between all isotope data with the identified dietary groups (Fig. 5A) reveals a significant isotopic difference between individuals on a C_3_ diet and C_4_ consumers for most isotopes (*P* values listed in Table 2 for each isotopic system). The difference in carbon isotopes from the diverse tissues is intrinsic to this group’s definition. No difference can be observed between dietary groups with respect to δ^18^O_DW_ values. The collagen δ^15^N values tend to be slightly more elevated in the tissues of C_4_ consumers compared to non–C_4_ consumers, yet this is only weakly significant for the petrous bones (*F* = 7.4, *P* = 0.002 at all sites) and not statistically significant for the bone and dentin data (*P* > 0.1) (Fig. 5A and Table 2). However, it is notable that individuals on a C_3_ diet have significantly lower ^87^Sr/^86^Sr values and more elevated [Sr] values in their enamel compared to individuals with isotopic evidence for C_4_ intake (Fig. 5A and *F* and *P* values in Table 2). To complete this observation, it is noteworthy that individuals showing dental enamel ^87^Sr/^86^Sr or δ^18^O_DW_ values falling outside the expected local range of their respective sites (see definitions in Materials and Methods) are also significantly associated with elevated δ^13^C values typical for a C_4_ intake, regardless of the investigated tissue (Fig. 5B and see *F* and *P* values in Table 2). The results at the site level, however, underlines the great diversity of combinations between dietary groups and Sr or O isotopic diversity at the site level (fig. S5).

**Table 2.** Detailed results of the one-way ANOVA tests. For all samples dated between around 1700/1600 and 1000 BCE, the tests determine the significance of isotopic variability observed between individuals with various diets or various δ^18^O and ^87^Sr/^86^Sr backgrounds and between adult males and adult females. In each case, the first round of tests includes all sites from this period and the second option focuses only on sites with isotopic evidence for C_4_ consumption. The box plots that illustrate these isotopic distributions (based on all sites of this period) are available from Figs. 5 and 6.

One-way ANOVA tests (for individuals dated from ∼1700 to 1000 BCE)		Bone collagen δ^15^N values	Bone collagen δ^13^C values	Dentin collagen δ^15^N values	Dentin collagen δ^13^C values	Petrous collagen δ^15^N values	Petrous collagen δ^13^C values	Enamel δ^13^C values	Enamel δ^18^O_DW_ values	Enamel ^87^Sr/^86^Sr values	Enamel [Sr] values
C_3_ diet versus C_4_ consumption (all sites)	*F* value	2.1	221	0.3	195.8	7.4	110.3	108.6	2.1	6.9	12.1
	df	2	2	2	2	2	2	2	2	3	2
	Denominator df	87	87	89	89	45	45	52	52	96	40
	*P* value	0.1239	<2.2 × 10^−16^	0.7721	<2.2 × 10^−16^	0.002	<2.2 × 10^−16^	<2.2 × 10^−16^	0.1351	3.00 × 10^−4^	7.87 × 10^−5^
	Significance	Weak and not significant	Strong and significant	Weak and not significant	Strong and significant	Weak but significant	Strong and significant	Strong and significant	Weak and not significant	Weak but significant	Weak but significant
C_3_ diet versus C_4_ consumption (only at sites with C_4_ diet)	*F* value	1.3	79.4	0.3	46.2	0.7	25.3	53.7	2.3	1	1.5
	df	2	2	2	2	2	2	2	2	2	2
	Denominator df	54	54	40	40	19	19	40	40	41	28
	*P* value	0.331	<2.2 × 10^−16^	0.7578	4.04 × 10^−11^	0.498	4.43 × 10^−6^	4.63 × 10^−12^	0.1131	0.3903	0.25
	Significance	Weak and not significant	Strong and significant	Weak and not significant	Moderate and significant	Weak and not significant	Moderate and significant	Strong and significant	Weak and not significant	Weak and not significant	Weak and not significant
Inside versus outside ^87^Sr/^86^Sr range (all sites)	*F* value	3.6	28.5	3.5	44.2	24	33.8	11.9	2.3	50.7	37.5
	df	1	1	1	1	1	1	1	1	1	1
	Denominator df	70	70	87	87	22	22	53	53	98	41
	*P* value	0.06198	1.09 × 10^−6^	0.06	2.47 × 10^−9^	6.68 × 10^−5^	7.53 × 10^−6^	1.12 × 10^−3^	0.135	1.79 × 10^−10^	2.87 × 10^−7^
	Significance	Weak and not significant	Moderate and significant	Weak and not significant	Moderate and significant	Weak but significant	Moderate and significant	Weak but significant	Weak and not significant	Strong and significant	Moderate and significant
Inside versus outside ^87^Sr/^86^Sr range (only at sites with C_4_ diet)	*F* value	1.4	2.7	3.4	3.9	16.9	9.3	1.9	1.9	109.6	12.4
	df	1	1	1	1	1	1	1	1	1	1
	Denominator df	45	45	41	41	9	9	41	41	42	29
	*P* value	0.2386	0.1067	0.07144	0.0554	0.002593	0.01398	0.1705	0.1745	2.82 × 10^−13^	1.45 × 10^−3^
	Significance	Weak and not significant	Weak and not significant	Weak and not significant	Weak and nearly significant	Weak but significant	Weak but significant	Weak and not significant	Weak and not significant	Strong and significant	Moderate and significant
Males versus females (adults; all sites)	*F* value	0.5	1.4	1	2.2	0.2	0.4	2	1.3	0.4	0.3
	df	1	1	1	1	1	1	1	1	1	1
	Denominator df	78	78	78	78	39	39	50	50	86	38
	*P* value	0.4786	0.2398	0.3142	0.1428	0.6282	0.5473	0.1649	0.2675	0.5239	0.5682
	Significance	Weak and not significant	Weak and not significant	Weak and not significant	Weak and not significant	Weak and not significant	Weak and not significant	Weak and not significant	Weak and not significant	Weak and not significant	Weak and not significant
Males versus females (adults; only at sites with C_4_ diet)	*F* value	1.5	0.9	2.5	2.1	0.2	0.2	2.2	0.8	0	1.3
	df	1	1	1	1	1	1	1	1	1	1
	Denominator df	51	51	40	40	18	18	40	40	41	28
	*P* value	0.2312	0.3581	0.1241	0.1516	0.6617	0.691	0.1431	0.3641	0.8929	0.2662
	Significance	Weak and not significant	Weak and not significant	Weak and not significant	Weak and not significant	Weak and not significant	Weak and not significant	Weak and not significant	Weak and not significant	Weak and not significant	Weak and not significant

### Amelogenin protein analyses and sex determination

The biological sex of 35 adult and juvenile individuals was successfully identified (fig. S6 and data S1) using an untargeted paleoproteomic approach. Results showed concordance between sex identification based on osteomorphology, ancient genomics, and paleoproteomics for all individuals except MIL1_233 (data S1). The MIL1_233 individual was previously identified as male based on both ancient DNA (aDNA) analysis (*38*) and osteomorphology. However, in this paleoproteomic study, no sex-specific peptides corresponding to the AMELY protein was observed. On the basis of this result, we would classify the MIL1_233 individual as female by paleoproteomics. Buonasera *et al.* (*61*) also reported sex identification conflicts occurring with both aDNA and paleoproteomic biological methods. As in Buonasera *et al.* (*61*), our MIL1_233 individual does not show a decrease in paleoproteomic signal [log_10_(AMELX); fig. S6]. Paleoproteomic and aDNA methods are both robust. In this study, we favored the detection of the Y chromosome during aDNA analyses, and MIL1_233 is interpreted as male throughout this study.

### Relationship among diet, sex, and age

**Fig. 6.**
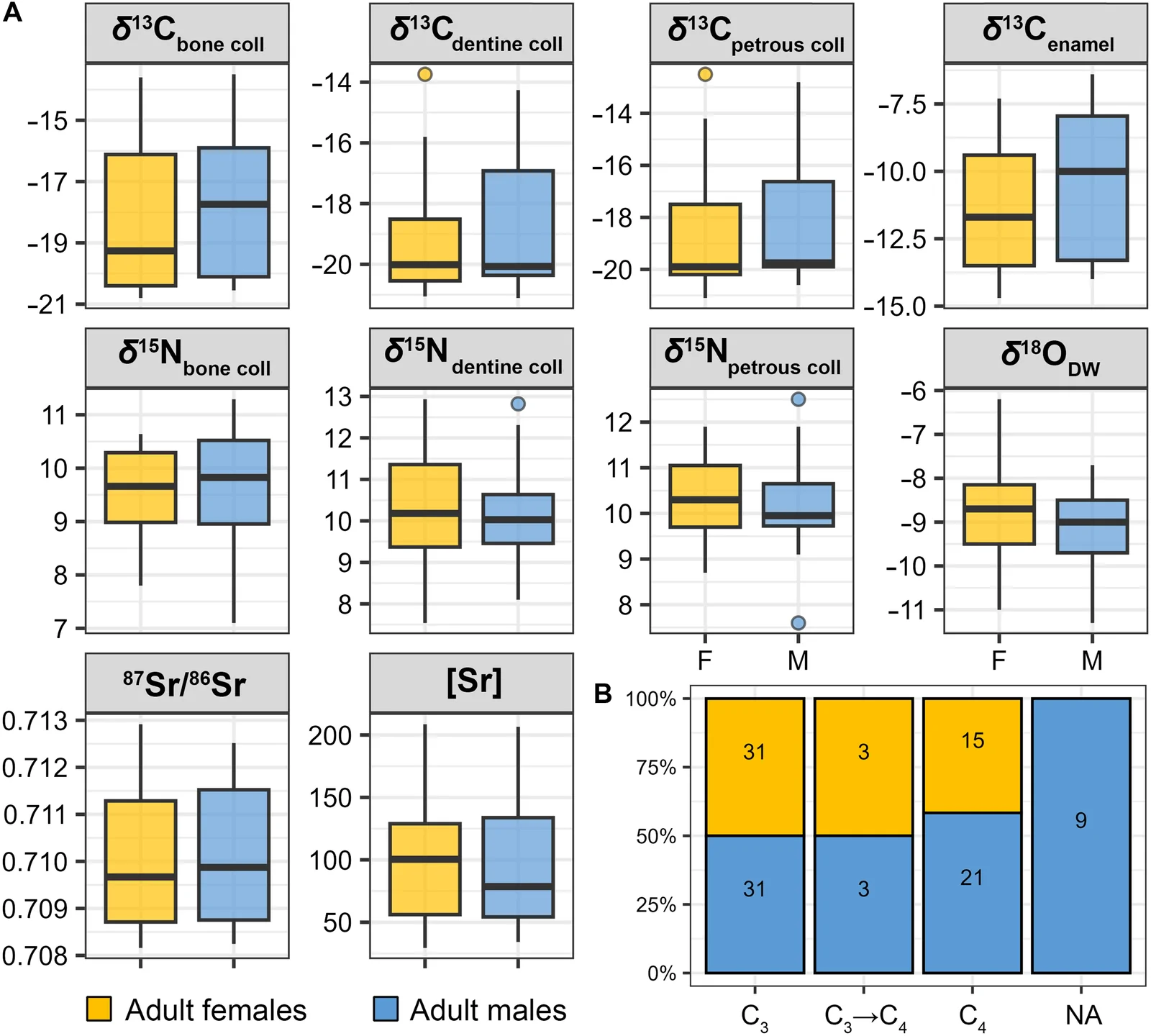
Comparison of dietary habits and isotopic compositions between adult males and females for all individuals dated to ∼1700/1600 to 1000 BCE. The sex identification is based on new paleoproteomic and/or published paleogenetic data (*16*, *38*, *39*) for slightly more than half of this sample, while the other half is based on osteological morphometry (see details in data S1, fig. S7, and Materials and Methods). (**A**) Box plots showing isotopic differences between adult male (M) and female (F) individuals. Results of the statistical tests including adult males and females only are listed in Table 2. (**B**) Counts and proportions of adult males and adult females from all sites within the identified dietary categories.

**Fig. 7.**
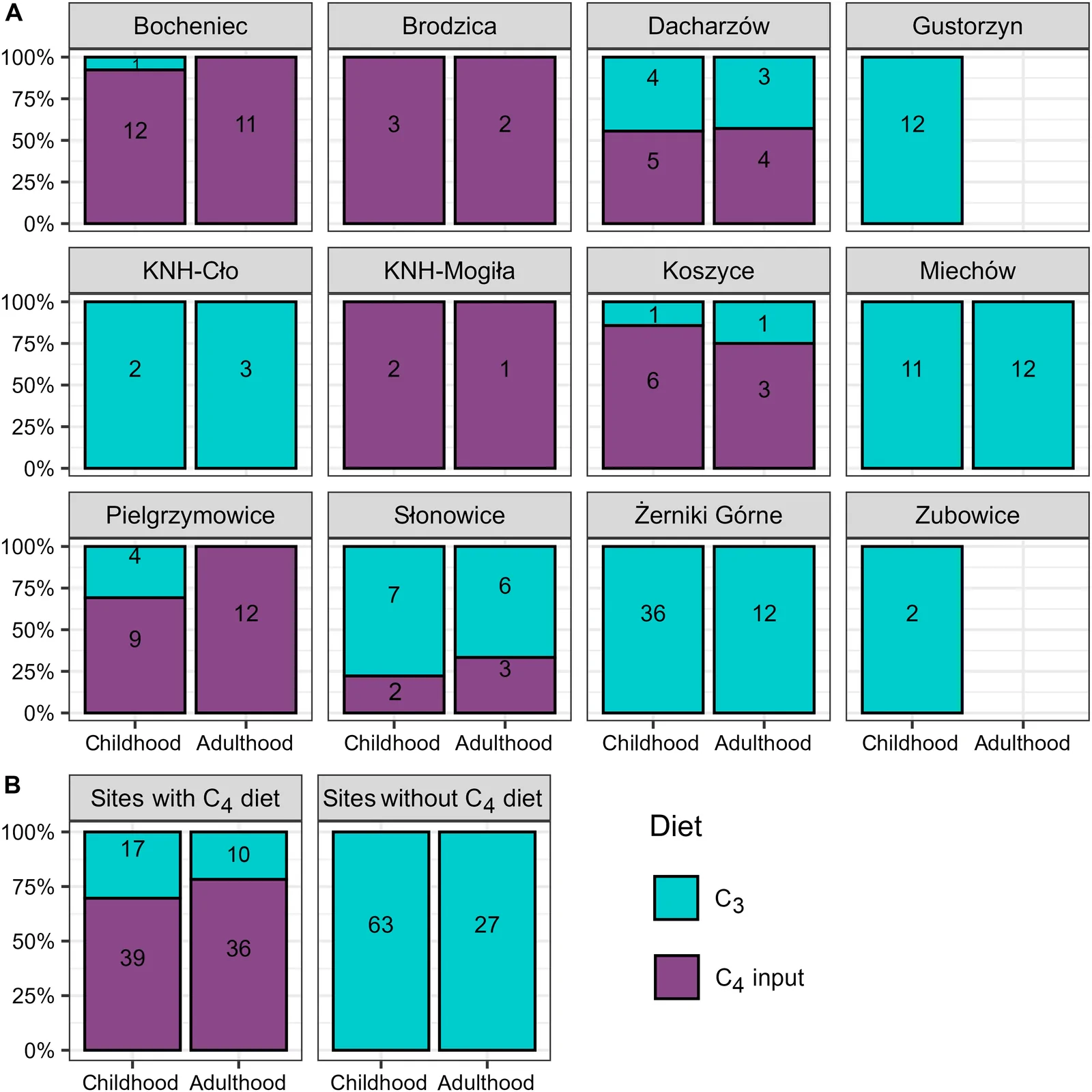
Comparison of dietary habits and isotopic compositions between childhood and adulthood of the various individuals dated to ∼1700/1600 to 1000 BCE. (**A**) The results are depicted by site. (**B**) The results are shown separately for sites with and sites without carbon isotopic evidence for millet consumption. In each case, the life stage is determined from both the age at death and the analyzed tissue (see details in data S1). Not all individual provided data for both childhood and adulthood. The total number of analyzed individuals with dietary data (i.e., δ^13^C data) from each site is available from Table 1. The evolution of collagen δ^13^C data over the lifetime of each individual at each site is depicted in fig. S8.

When considering all individuals from the research area dated to after the first evidence of millet consumption (i.e., after 1600 BCE), there is no statistically significant difference between males and females in terms of isotopic composition (Fig. 6A and see the related *P* values in Table 2) or C_4_ consumption (Fig. 6B). This is largely confirmed at the site level (fig. S7). With our multitissue isotopic approach, it is furthermore possible to verify whether millet was preferably consumed during specific life stages. At all sites, 67% (*n* = 80) of the individuals with childhood dietary data showed a C_3_ diet in childhood, against 33% (*n* = 39) showing evidence for millet consumption. Conversely, 50% (*n* = 37) of the individuals with adulthood dietary data were on a C_3_ diet during adulthood, and the other half were consuming millet. Millet was therefore not preferably consumed during specific life stages (Fig. 7A). When considering only sites with isotopic evidence for C_4_ consumption, we observe the same proportion of individuals on a C_3_ diet (i.e., around 22 to 30%) or respectively with millet consumption (i.e., 70 to 78%) in both childhood and adulthood (Fig. 7B), stressing again that there were no dietary differences between life stages.

## DISCUSSION

### A revised chronology of millet introduction in Central Europe

In Europe, the earliest archaeobotanical millet macroremain so far is dated to ∼1546 ± 44 cal BCE and was found in Vinogradnyi Sad, southern Ukraine (*5*). The spread model by Filipović *et al.* (*5*) further suggests that millet had reached southern Poland in the 14th century cal BCE, with the earliest directly dated macrobotanical evidence of broomcorn millet in (southern) Poland dating back to 1303 ± 59 cal BCE at Lipnik and to 1305 ± 55 cal BCE at Maszkowice (*5*, *62*), which is around 240 years later than in Ukraine. However, recent stable isotope studies revealed that the incorporation of millet in human diet was already isotopically visible from 1457 ± 36 cal BCE onward at Pielgrzymowice, southeastern Poland (*16*). This is contemporaneous with the earliest isotopic evidence from Kordyshiv, western Ukraine, showing the earliest C_4_ consumers by 1432 ± 40 cal BCE (*13*, *16*). With our multitissue isotopic approach combined with new and existing ^14^C dates (*16*, *38*), we push back the earliest date of millet consumption in Europe by more than a century (Table 1 and Fig. 3), reaching 1588 ± 48 cal BCE (Table 1 and see MIL1_234 on Figs. 2 and 3). This predates the evidence from radiocarbon-dated macroremains of Central Europe (*3*, *5*) but is broadly contemporaneous with the earliest radiocarbon-dated macroremains reported from southern Ukraine (*5*), suggesting an earlier spread from Eastern to Central Europe than previously expected.

Another individual from the same site (MIL1_233) shows an early evidence for millet intake, 1468 ± 31 cal, revealing a sporadic consumption before millet was more widely adopted as a staple crop by around 1400 BCE (Figs. 2 and 3 and data S2). The progressive character of early millet consumption suggests that the crop was already known and available in the region at least one century before it became a staple. This confirms tendencies observed from previous studies (*4*, *14*, *18*) and demonstrates the need to combine several lines of evidence to disentangle millet spread from its adoption as backup or staple crop. Moreover, people identified with a C_3_ diet in childhood before changing to a mixed C_3_-C_4_ diet in adulthood were identified at all sites with evidence for millet consumption and were often the chronologically oldest individuals with evidence for millet consumption at their site (except at Dacharzów and Koszyce; see Fig. 2). Beyond individuals changing diet, we observe at four of seven sites with isotopic evidence for millet consumption that the role of millet in some individual’s diet increased over their lifetime (data S1). This shows that our multitissue and multiproxy approach enabled us to seize the first gradual introduction of millet and then the actual moment of its adoption as staple crop within the investigated communities.

Radiocarbon dates coupled to stable carbon isotope data moreover highlight that C_3_ diets are still attested after the arrival of millet in the region. This occurs not only at sites at which the whole community remained on a C_3_ diet (e.g., Żerniki Górne, Miechów, and Gustorzyn), despite their location in direct vicinity to sites with evidence for millet consumption [see (*63*) for further regional comparisons around Gustorzyn], but also at sites with isotopically identified millet consumers (e.g., Dacharzów and Koszyce) (Figs. 1 and 2). Consequently, millet introduction within a community or a region did not imply a systematic change of diet for the whole group. In their recent study, Pospieszny *et al.* (*16*) associated the presence or absence of millet consumption with a site location in the upland or lowland, respectively. Hence, a specialized economy with access to different parts of the landscape is one explanation for these dietary differences between sites. However, cultural choices, culinary traditions, or individual preferences cannot be excluded, especially to explain contemporaneous dietary differences within sites (Fig. 2 and figs. S1 and S2). This was suggested not only by Pospieszny *et al.* (*16*) for this region but also by other authors in other regions and periods (*22*, *64*–*67*).

A rapid decrease in millet consumption after the peak of its adoption is attested for example in Germany (*22*). However, the results from southeastern Poland do not provide a similar pattern at this stage. The only individual showing decreasing millet inputs over his lifetime (MIL1_230 at Pielgrzymowice) is not radiocarbon dated. It is therefore impossible to determine whether this male individual belongs to the latest chronological phase of this site. Any other reason may have caused such a reduction of millet input in his diet. As for the few individuals dated to the Late Bronze Age or Early Iron Age (*n* = 4), these are one non–millet-eater from Żerniki Górne, where millet apparently was largely not adopted, and three from Miechów, where, on the contrary, millet seems to just start appearing toward these later phases (*53*). However, each region of Europe evolved differently (*22*, *24*, *64*, *65*, *68*–*70*), and more data are needed to determine whether the evolution of millet consumption after the Middle Bronze Age in our research area.

### Land-use shift for millet cultivation

Investigating the introduction of millet into Central Europe raises the question of its potential demic diffusion (*71*). The significant differences in ^87^Sr/^86^Sr values and [Sr] between people on a C_3_ diet and C_4_ consumers with isotopic evidence for C_4_ intake (Fig. 5A and *F* and *P* values in Table 2) and, in particular, the association between C_4_ consumption and unexpected ^87^Sr/^86^Sr or δ^18^O_DW_ values (Fig. 5B) may at first give the impression that millet was primarily consumed by people who originated from another geographical area (Fig. 4 and fig. S4), apparently supporting the hypothesis of a demic diffusion. Another line of evidence may be the outlier genetic background of the six individuals buried in grave 669 at Pielgrzymowice, matching their outlier ^87^Sr/^86^Sr values (*38*). However, they are not the earliest millet consumers nor the earliest ^87^Sr/^86^Sr outliers from this site. Similarly, the possibly earliest individual consuming millet at Słonowice has outlier ^87^Sr/^86^Sr values but was not radiocarbon dated (Fig. 4).

It is moreover noteworthy that the earliest millet consumers identified in the region were people with local ^87^Sr/^86^Sr values, who had a clear C_3_ diet during their childhood and adopted millet only later in their lives (e.g., MIL1_234 and MIL1_233 on Figs. 2 and 4). At the site scale, Bocheniec, Pielgrzymowice, and Słonowice show this pattern of the earliest isotopic evidence for millet consumption found in individuals who changed from a C_3_ diet during childhood to C_4_ inputs during adulthood (Fig. 2). Except at Brodzica (*n* = 3), the first individuals buried at each site were systematically on a C_3_ diet (Fig. 2). This continuity between people with and people without millet consumption is further attested by their shared Trzciniec archaeological background (∼1800 to 1200/1100 BCE) (*43*, *72*, *73*) and overall lack of outstanding genetic outliers (*38*, *74*), except for the abovementioned individuals in grave 669 at Pielgrzymowice (*38*). It implies that millet was adopted in the first place by local communities, who were originally on a C_3_ diet, unless the first generation of “nonlocal” millet consumers is missing from our sample, which cannot be the main argument due to the obvious lack of evidence.

In addition, the results of the KDE models on radiocarbon dates demonstrate that neither the millet consumers nor the individuals with outlier ^87^Sr/^86^Sr values represent a single chronological event, be it at the regional or at the site level (data S2). Together with the great diversity of scenarios attested at each site (Fig. 4 and fig. S5), this contradicts the hypothesis of a nonlocal C_4_ community bringing millet to southern Poland in one migration wave, which would have been otherwise the only logical explanation for a general nonlocal origin of all individuals buried at sites with isotopic evidence for millet consumption from 1500 BCE. In contrast, it is unlikely that people (usually consuming millet) would keep arriving from another region over time to be buried in these same places, without any local individual, not even the offspring of the first generation of newcomers, to be buried there.

Instead, the overall shift in ^87^Sr/^86^Sr and [Sr] values widely observed from around 1500 BCE between individuals consuming important amounts of millet and people on a C_3_ diet (Figs. 4 and 5 and figs. S4 and S5) more probably reveals a shift in subsistence strategies. Because millets are more resistant to salinity, poor soils, and/or dry conditions than C_3_ crops, they could be grown in places unsuitable for a C_3_-based agriculture (*75*, *76*), introducing new or otherwise underrepresented Sr isotopic composition in the body tissues of millet consumers. Geological, hydrological, and bioarchaeological studies [e.g., (*54*, *77*–*81*)] and the global model for bioavailable Sr (*59*) highlight the great Sr isotopic variability in the region, which confirms that using different areas for the cultivation of millet compared to the C_3_ crops is a plausible explanation for the observed disparities in ^87^Sr/^86^Sr and [Sr] values between C_3_ and C_4_ consumers within the same region. Several studies using multiproxy (isotopic) data from other periods and regions recently drew similar conclusions, favoring a land-use and local subsistence shift instead of a demic diffusion model (*18*, *20*, *70*), in particular, the study in Late Bronze Age Germany that exhibits the exact same patterns in archaeological, genetic, and Sr and C isotope data (*19*), supporting our interpretation.

Another or complementary option to explain the access to new areas for millet cultivation (as reflected from the Sr isotope data) is that a mobile pastoralist economy became the rule when millet was established as a staple crop among many communities in southern Poland. Its short vegetation period, high resistance, low required cultivation effort, and high nutritious status make it an ideal crop for this kind of subsistence (*2*, *10*, *14*). The lower [Sr] values may, in this case, rather reflect the more important role of animal products in the diet of “pastoralist” C_4_ consumer (*82*, *83*) compared to farmers on a C_3_ diet. The differences in δ^15^N values between C_3_ and C_4_ consumers are less obvious than for Sr and mostly not statistically significant (*F* and *P* values in Table 2), yet the tendentially more elevated δ^15^N values in C_4_ consumer collagen tissues might support this hypothesis (Fig. 5A). Either way, the adoption of millet as staple facilitated, required, or was triggered by an important shift in subsistence strategies in which access to previously uncultivated areas became possible.

### The sociocultural background of millet introduction

Millet was introduced in southeastern Poland by the time communities of the Trzciniec Cultural Circle (∼1800 to 1200/1100 BCE) (*72*, *84*) were established as the local population (*38*, *72*). In this long-lasting Trzciniec context, the only notable archaeological difference between C_3_ consumers and millet-eaters, namely, the absence of millet-eaters among the burials under barrows, reflects a chronological bias [e.g., at Dacharzów; see (*40*)]. Similarly, the only two millet consumers from Słonowice were buried together in the same grave (feature 12/2004), but they also represent a later chronological phase (Fig. 2 and data S2).

At Koszyce, Bocheniec, and Pielgrzymowice, millet consumers are moreover buried in collective graves together with non–millet-eaters (or with individuals who originally had a C_3_ diet in their childhood before changing diet by integrating millet) and without evidence for diverging burial practices (*43*, *49*, *73*, *84*). The great diversity in culinary habits can therefore be put into perspective with the particularly diverse cultural practices archaeologically attested for this period (*72*, *84*). In the settlements, this includes a network of large central settlements, smaller filial sites, and temporary camps, while within the burial practices, this includes both inhumation and cremation, both barrow and flat graves, the perpetuation of individual burials despite the new predominant collective and multiple graves, the emergence of human-animal burials in domestic pits or sacrifice contexts, and the diversity of burial places within the settlement or on cemeteries and within burial or domestic pits (*43*, *72*, *84*). In terms of subsistence practices, the Trzciniec groups are characterized by more flexible economic strategies than previous cultures in the region, since they settled on fertile chernozems developed over loess, as well as on less productive soils, and they combined cereal agriculture, animal husbandry, hunting, gathering, and the exploitation of natural resources such as salt deposits (*84*). Genetic and material influences from the South and specifically from the Carpathian Basin complete this multifaceted picture (*16*, *38*, *74*, *84*).

From the archaeological perspective, the burial practices and evidence for elaborated and lavish graves at Dacharzów (*40*) are furthermore very similar to what is attested at Żerniki Górne (*85*), despite the lack of isotopic evidence for millet consumption in the latter. This stresses the lack of relationship between social status or burial practices and millet adoption or rejection, as expected from their largely shared archaeological (*55*, *72*) and genetic background (*38*). Similarly, the lack of difference in dietary habits between males and females (Fig. 6) or between life stages such as childhood versus adulthood (Fig. 7 and fig. S8) agrees with the lack of difference in burial practices between males and females and between adults and subadults (*55*, *72*, *84*). The only difference that remains at the regional level is at the ecological level, as sites with no carbon isotopic evidence for millet consumption are preferably located in the lowlands, whereas sites with evidence for C_4_ diets are located on the uplands (*16*). This suggests that the adoption or rejection of millet as staple crop was primarily related to site-specific environmental constraints and affordances, while its consumption was a large-scale phenomenon that touched all parts of the society in communities where it was introduced.

Consequently, there is no strong evidence supporting the hypothesis that millet was consumed by one specific part of the society or that it was brought in one wave to southwestern Poland by millet-consuming migrants shortly after 1700/1600 BCE. Instead of a demic diffusion model, the multiproxy isotopic and radiocarbon data suggest a progressive introduction of millet, after which the adoption of this crop as staple can be associated with an important change in subsistence strategies involving new land-use opportunities. It is possible that the use of previously uncultivated areas was needed to cope with changing climatic conditions (*86*, *87*) or as a response to demographic pressure (*16*).

### Limitations and perspectives

This study reveals that a multitissue and multi-isotopic approach coupled to radiocarbon dates offers the possibility to revise the chronology of important dietary and/or subsistence shifts in past societies. However, this implies that dietary changes are possibly still undetected for the individuals for which only childhood dietary data are available in this study (e.g., only from dental or petrous bone tissues). We therefore encourage future research to undertake these multitissue and multiproxy studies to more accurately investigate the evolution of subsistence strategies and the multifaceted complexity of the human past.

Furthermore, this multitissue approach revealed the gradual aspect of millet introduction not only into Bronze Age communities but also into individual’s diet. When considering the individuals who changed from a C_3_ to a mixed C_3_-C_4_ diet over life, the multitissue carbon isotope analyses often presented a progressive increase in (collagen) δ^13^C values over life, e.g., for MIL1_309 at Bocheniec, MIL1_321 and MIL1_334 at Pielgrzymowice, MIL1_315 at Koszyce, and MIL1_331 at Słonowice (Fig. 2 and fig. S2). This might mean that millet was first introduced into these individual’s diet in small quantities, before this dietary input became substantial enough to be isotopically visible or before the tissue remodeling turnover enabled its full record. At sites with carbon isotopic evidence for millet consumption, some individuals interpreted as being on a C_3_ diet moreover show δ^13^C values that are closer to the local δ^13^C threshold than individuals on a C_3_ diet from sites where no millet consumption is isotopically attested (Fig. 2 and figs. S1 and S2). Although a certain degree of carbon isotopic variability can be expected both within individuals on a C_3_-based diet and within individuals on mixed C_3_-C_4_ diets, for example, due to intrinsic measurement errors (*88*), due to microscale variability in environmental and climatic settings (*89*, *90*), due to the stable carbon isotopic diversity among different food from C_3_ ecosystems (*27*, *91*–*93*), or for physiological and metabolic reasons (*94*–*96*), this multitissue approach also suggest that indirect, periodic, particularly small, or gradually increasing C_4_ inputs may be identified from collagen and enamel δ^13^C values that are still considerably below local thresholds for C_4_ diet identification (Fig. 2, figs. S1 and S2, and see also Materials and Methods) (*30*). This shows that combining a multitissue and a large-scale diachronic approach increases the chances to identify millet consumers, even without substantial inputs or despite body tissue remodeling turnover rates.

We further stress the urgent need to create a robust Sr isoscape for the region, which would help to consolidate the hypothesis of a land-use shift instead of a demic diffusion model. This will be possible with the systematic sampling and analysis of samples suitable for site-specific and regional ^87^Sr/^86^Sr baseline determinations (*34*, *97*) coupled with multivariate environmental analyses (*35*).

More research is also needed to disentangle simultaneity from causality when comparing millet adoption with climatic or societal changes. It is therefore not yet possible to state whether the adoption of millet as staple crop triggered the land-use shift by offering new opportunities or whether the need for optimized subsistence and land-use strategies (e.g., as a response to climate change, demographic pressure, or other factors) implied the necessity to adopt a new staple crop (millet) that offered solutions to cope with the new challenges. Improving the chronological model with more radiocarbon dates and integrating environmental and climatic variables (*66*, *98*, *99*) would offer further insights on this topic.

Plants usually exhibit the highest Sr concentration along the food chain (*82*, *83*). However, on the basis of the low [Sr] in millet consumer’s enamel (Fig. 5A), we may assume that millets have lower [Sr] than C_3_ crops. To the best of our knowledge, no data are currently available to confirm or reject this hypothesis. However, if this were true, then the dietary resource that predominantly affects the Sr isotope ratios in the skeletal tissues of millet consumers would no longer be the crops, as it is for C_3_ consumers, but some other food resource. Given the ^87^Sr/^86^Sr variability that can be expected from diverse food resources in southern Poland, the replacement of C_3_ crops by millet would, in this case, have affected the enamel’s ^87^Sr/^86^Sr values without any mobility or land-use shift. More fundamental research that verifies the Sr concentration of millets under various growing conditions and compares it with [Sr] in C_3_ plants is, however, required to determine whether this hypothesis can be securely rejected or should be considered in the interpretation of the data. A land-use shift from around 1500 BCE, including the cultivation of previously inaccessible or unused ecological areas (with or without an association to a pastoralist economy), remains the preferred explanation for our results in the current state of research.

## MATERIALS AND METHODS

### Experimental design

To complete existing datasets from selected Bronze Age sites in Poland (*16*, *40*, *53*–*55*) and enhance the current stage of knowledge, 152 individuals from 13 Bronze Age archaeological sites (Fig. 1 and Table 1) were selected for multiple isotope analyses.

Whenever possible, dentin and bone collagen samples were collected for each individual to perform stable carbon (C) and nitrogen (N) isotope analyses (producing δ^13^C and δ^15^N data), which are used to identify millet consumption (see detailed description below) at different life stages, i.e., during childhood from the dentin and during the last years of life from the bone. More precisely, the earliest phase of the childhood (in utero until maximum 10 years of age) is reflected in the deciduous teeth and the permanent first permanent molars, canines, and incisors; the middle phase of the childhood (between one and maximum 16 years of age) is reflected by the permanent second molars and premolars; and the latest phase of the childhood (from the 7th year of life to early twenties) is represented by the third permanent molar, with the dentin forming each time more quickly than the enamel (*57*). In contrast, bones may reflect between the last 5 years of life (e.g., ribs) and the last decades of life (e.g., femur) (*56*).

Early childhood data were furthermore available for some individuals from published δ^13^C and δ^15^N data derived from their previously analyzed petrous bones (*16*). Whenever available, the dental enamel of these individuals was further sampled for C, oxygen (O), and strontium (Sr) isotope analyses (producing δ^13^C, δ^18^O, ^87^Sr/^86^Sr, and [Sr] data). The enamel δ^13^C is a complementary information to the collagen values regarding dietary habits, as it reflects the whole diet (and not only its protein part) and is thus more sensitive to the plant-based food included in the diet (*28*). The δ^18^O, ^87^Sr/^86^Sr, and, to some extent, [Sr] data inform about the geographical origin of the food ingested during the formation of the teeth and can, in turn, inform about mobility patterns (*33*). The enamel of the selected individuals was moreover analyzed for amelogenin protein composition to determine the biological sex, completing information from previous genetic (*38*) and osteological analyses.

For each individual, we therefore produced and collected information about dietary habits during different life stages to distinguish millet consumers from non–millet consumers and to identify potential dietary changes over their lifetime. Most investigated individuals derive from multiple burials in which skeletons were more or less disarticulated and mixed. However, to the best of our knowledge and expertise, the bones and teeth presented as belonging to one single individual belong together (*48*). Intensive aDNA investigations would be needed to confirm this, which would have spread the scope and budget of this study. Combined with the abovementioned existing data from those sites (*16*, *40*, *53*–*55*), this results in a series of published and newly generated multiple stable isotope data (of C, N, O, and Sr) on diverse skeletal tissues (dental enamel and dentin, petrous bone, and bone collagen) from a total of 192 archaeological humans (data S1).

### Archaeological skeletal material and ethics statements

The skeletal material originated from various storage institutions in Poland (see data S1 and the “Data, code, and materials availability” statement). Access to the material was provided following the required ethical and scientific integrity procedures and in full collaboration with local stakeholders (see text S1 for more details on the provenance of the material). No living relatives are known for these archaeological communities, and, therefore, no ethical issues exist related to excavation, conservation, analysis, and publication. The sampling strategy for destructive analyses was furthermore adapted to preserve the material as much as possible, for example, by using samples from the same bones and teeth for various analyses and by completing existing datasets.

### Stable isotope analyses on collagen

In total, 139 dentin and 52 bone collagen samples deriving from a total of 138 individuals were analyzed to complete existing isotope datasets from Bronze Age Poland. Bone and dentin samples were collected and prepared in the laboratory facilities of the Bioarcheology Research Center at Vilnius University, following the procedure described by Richards and Hedges (*100*). Cleaned bone pieces of around 200 mg were demineralized at room temperature in 7 ml of a 0.5 M HCl solution changed every 24 to 48 hours over a week. The demineralized residue was rinsed six times with ultrapure water, then placed in 7 ml of a pH 3 HCl solution, and then gelatinized for 48 hours in the oven at 75°C. The clean solution was then placed in the freezer overnight and then freeze dried for up to 48 hours to extract collagen. The same procedure was followed for dentin pieces after the collection of their dental enamel (see below) yet in 2 ml of a 0.5 M HCl solution for demineralization and in 2 ml of a pH 3 HCl solution for gelatinization. All bones and 122 of 137 dentin samples provided collagen. Between 0.70 and 0.95 mg of the extracted bone or dentin collagen was placed in a tin capsule for stable C and N isotope analysis.

Stable isotope ratio measurements were performed via continuous flow (elemental analysis) isotope ratio mass spectrometry (MS) (CF-EA-IRMS) at the Center for Physical Sciences and Technology (FTMC; Vilnius, Lithuania) using a Flash EA1112 C-N-S elemental analyzer (Thermo Fisher Scientific, Bremen, Germany) coupled to Thermo Delta V isotope ratio mass spectrometer (Thermo Fisher Scientific, Bremen, Germany). Isotope ratios were expressed using the widespread δ notation, in per mil and relative to the international references VPDB (for carbon) and atmospheric air (for nitrogen). The accuracy was determined by repeated measurements of the international reference materials IAEA-600 (caffeine, with a mean δ^13^C of −27.77 ± 0.04‰), USGS24 (graphite, with a mean δ^13^C of −16.05 ± 0.04‰), IAEA-N-1 (ammonium sulfate, with a mean δ^15^N of 0.4 ± 0.1‰), and IAEA-N-2 (ammonium sulfate, with a mean δ^15^N of 20.4 ± 0.1‰). Caffeine (Sigma-Aldrich; with a mean δ^13^C of −32.68 ± 0.15‰ and a mean δ^15^N of −2.9 ± 0.2‰) was used as a secondary analytical standard. The precision [u(Rw)] was determined to be ±0.12‰ for δ^13^C and ±0.18‰ for δ^15^N on the basis of repeated measurements of calibration standards and sample replicates. The total analytical uncertainty was estimated to be ±0.16‰ for δ^13^C and ± 0.20 for δ^15^N.

Most stable isotope data from dentin and bone collagen samples met the quality criteria (*101*, *102*), except four dentin samples (MIL1_241, MIL1_248, MIL1_258, and MIL1_280) with too elevated C/N atomic ratios. These were therefore excluded from the study and are marked in red in data S1 [see also (*52*)].

### Isotope analyses on enamel carbonate

The enamel of 133 individuals was successfully analyzed for ^87^Sr/^86^Sr values. In addition, because the Sr isotope analyses were carried out in two different laboratories, i.e., at the Isotope Laboratory of the Adam Mickiewicz University in Poznań, Poland and at the Archaeology, Environmental Changes and Geo-Chemistry (AMGC) Research Group, at the Vrije Universiteit Brussel (VUB), in Brussels, Belgium, the enamel of 21 individuals originating from the various sites was analyzed in both laboratories to make sure that their results are comparable and could be used together in this study. Moreover, 56 of these enamel samples were analyzed for δ^18^O and δ^13^C isotopic composition at the AMGC, VUB.

For strontium isotope analyses carried out in the Isotope Laboratory at the Adam Mickiewicz University in Poznań, Poland [*n* = 110 teeth; see data S1 and (*52*)], the mechanically isolated enamel was first cleaned in the ultrasonic bath in ultrapure water to remove sediment particles. Afterward, about 10 mg of powdered enamel was treated sequentially with 2 ml of 0.1 N ultrapure acetic acid (five times) to eliminate the diagenetic Sr contamination, according to the procedure described in (*103*). Subsequently, the samples were dissolved on a hot plate (∼100°C, overnight) in closed perfluoroalkoxy (PFA) vials using 1 N HNO_3_. The miniaturized chromatographic technique developed by Pin *et al.* (*104*) and modified by Dopieralska (*105*) was applied for Sr separation. Sr was loaded with a TaCl_5_ activator on a single Re filament and analyzed in dynamic multicollection mode on a Finnigan MAT 261 mass spectrometer. Samples were corrected for mass-dependent fractionation by internal normalization to a fixed ^86^Sr/^88^Sr of 0.1194 using an exponential mass bias law (*106*, *107*). During this study, the National Institute of Standards and Technology (NIST) standard reference material SRM 987 Sr standard yielded ^87^Sr/^86^Sr = 0.710218 ± 11 (2σ, *n* = 14). The measured ratios were normalized to a nominal value of 0.710248 for the standard NIST SRM 987. Total procedure blanks were less than 70 pg.

Besides, 57 teeth were analyzed for C, O, and/or Sr isotopic composition carried out at AMGC, VUB [data S1 and (*52*)]. The required enamel powder was sampled in the laboratory facilities of the Bioarcheology Research Centre at Vilnius University. The teeth were cleaned in ultrapure water using an ultrasonic bath. Once dry, a first enamel layer was removed before sampling around 30 mg of the required enamel powder using a handheld Dremel with a diamond drill headpiece and taking samples across the whole length of the crone. At AMGC, VUB, a pretreatment procedure (*108*) using 2 ml of 0.1 M unbuffered acetic acid for <30 min was applied to remove potential contaminants. The treatment was followed by a triple rinsing and then drying of the samples (*109*). The exact laboratory workflow for C, O, and Sr isotope analyses is available from Griffith *et al.* (*109*).

A Nu perspective IRMS with a NuCarb carbonate preparation device was used for C and O isotope analyses. Repeated analyses of the International Atomic Energy Agency calcite standard IAEA-603 produced mean values of 2.5 ± 0.1‰ for δ^13^C and −2.4 ± 0.1‰ for δ^18^O (*n* = 41, 1 SD). Measurements of the calcium carbonate standard NBS18 (*n* = 20, 1 SD) yielded −5.1 ± 0.1‰ for δ^13^C and −23.0 ± 0.2‰ for δ^18^O. Analyses of the calcite standard IAEA-CO-8 gave average values of −5.8 ± 0.1‰ for δ^13^C and −22.7 ± 0.3‰ for δ^18^O (*n* = 39, 1 SD). For the internal calcined bone standard CBA (*n* = 13, 1 SD), the measured values were −14.5 ± 0.4‰ for δ^13^C and −10.9 ± 0.3‰ for δ^18^O.

Strontium was extracted and purified following the methods of Snoeck *et al.* (*110*) and Gerritzen *et al.* (*111*). ^87^Sr/^86^Sr and [Sr] were both measured on a Nu Plasma 3 MC-ICP Mass Spectrometer (PD017 from Nu Instruments, Wrexham, UK) at AMGC, VUB. Strontium concentrations of bone samples were obtained by comparing the ^88^Sr voltage of each sample to the ^88^Sr voltage of a 30–part per billion [Sr] solution of NIST SRM987 (*112*). A simple rule of three was then applied to obtain the [Sr] of the samples with the precision and accuracy of the [Sr] analyses validated against certified standards. During this study, repeated measurements of NIST SRM987 returned ^87^Sr/^86^Sr = 0.710249 ± 0.000024 (2 SD for 15 analyses), which is consistent with the mean value of 0.710252 ± 0.000013 (2 SD for 88 analyses) obtained by thermal ionization MS instrumentation (*113*). Measurements were normalized using a standard bracketing method with a NIST SRM987 value of ^87^Sr/^86^Sr = 0.710248 (*113*). Procedural blanks were considered negligible [total Sr (V) of max 0.02 versus 9 to 10 V for analyses; i.e., ≈ 0.2%]. For each sample, the ^87^Sr/^86^Sr value is reported with a 2 SE error (absolute error value of the individual sample analysis − internal error). Matrix-matched standards were processed and measured in the same manner as samples. NIST SRM1400 bone ash returned an ^87^Sr/^86^Sr of 0.713112 ± 0.000032; 2 SD; [Sr] = 222 ppm ± 5% relative standard deviation (RSD); *n* = 14. This is in agreement with previously published values of ^87^Sr/^86^Sr = 0.713120 ± 0.000033 (2 SD; *n* = 6) (*114*).

The 21 dental enamel samples that were analyzed for Sr isotopic composition in both laboratories (AMGC at the VUB and Isotope Laboratory at the Adam Mickiewicz University in Poznań) to verify the comparability of the results yield a difference in ^87^Sr/^86^Sr values ranging from 2.83 × 10^−6^ to 0.0003 and averaging at 6.16 × 10^−5^ ± 7.69 × 10^−5^ (1 SD). This underlines the good adequation between both laboratories and the possibility to compare their results within this study, except for the MIL1_326, which shows an SD value of 0.0003 between both measurements. The difference may come from the fact that diverse parts of the enamel were analyzed in each laboratory, reflecting different mineralizing periods. However, because both values nevertheless fall within the local isotopic range, the interpretation as local for this sample was considered valid, and the sample was not excluded from the analysis.

### Identification of dietary habits

According to established threshold carbon isotope values, collagen δ^13^C values above −18.0‰ (*29*, *30*) and carbonate δ^13^C values above −10.0‰ (*31*, *32*) are considered as isotopic evidence for a C_4_ input into a C_3_ diet. However, the multitissue approach offered the possibility to balance the interpretation in case of contradictory results between different tissues and to identify particularly low C_4_ inputs by comparing data from various sites and individuals (figs. S1 and S2), with values very close to the established thresholds. In particular, enamel carbonate δ^13^C values are more representative for the entire diet including the plant part of the diet and collagen δ^13^C values are more affected by the protein part of the diet (*28*). The comparison between δ^13^C values of enamel and collagen (petrous bone, dentin, or other bones) therefore provides complementary insights to refine the interpretation in terms of dietary habits.

This applies for MIL1_335, who has a petrous bone collagen δ^13^C value of −17.1‰, previously interpreted as C_4_ intake (*16*). However, the three other tissues (dentin collagen, dental enamel, and bone collagen) reveal clear C_3_ signals. It is therefore possible that either the mother’s diet or factors other than a direct consumption of C_4_ food elevated the carbon isotope ratio during the formation of the petrous bone. The individual was therefore reinterpreted as being on an overall C_3_ diet.

Regarding MIL1_187, MIL1_235, MIL1_309, and MIL1_321, elevated δ^13^C values are found in their petrous bone and/or dentin collagen, whereas their dental enamels provide δ^13^C values below the usual threshold for C_4_ diet at −10‰. The contradiction between dentin collagen and dental enamel δ^13^C values may indicate that either (i) their diet changed toward a diet with (nearly) no millet input between the petrous and/or dentin formation and the completed mineralization of the enamel [which covers a gap of up to 8 years (*57*)] or (ii) the enamel δ^13^C value shortly below the threshold for C_4_ diet (Fig. 2) reveals a very low C_4_ consumption. The second option is considered more likely, revealing an increasing C_4_ input over their lifetime. This is particularly well exemplified for MIL1_309, for whom an M1, an M2, and a bone were analyzed. To keep a reasonable number of dietary categories (e.g., “C_3_ diet,” “C_3_ diet to C_4_ inputs,” and “C_4_ inputs”), the identification of diets reflecting an “increasing C_4_ consumption” is summarized under C_4_ inputs for the analyses.

As last example, the enamel δ^13^C value of mk57/MIL1_233 (−8.4‰) is above the threshold for C_4_ identification, whereas the dentin (−19.7‰) and petrous bone collagen (−19.9‰) δ^13^C values show a clearly C_3_ signals during childhood. The higher δ^13^C value measured in the costal bone collagen (−15.8‰) underlines a clear increase in C_4_ intake over life visible from collagen data. The enamel signal thus indicates that small and gradually increasing amounts of C_4_ foods were already incorporated into the diet by the time of the first molar’s mineralization in early childhood (*57*), while the collagen stresses an original diet based on C_3_ food.

### Oxygen isotope baseline in the region

To use the measured enamel carbonate δ^18^O values (versus VPDB) as reference for drinking water, these values were first converted into Standard Mean Ocean Water (SMOW) δ18O carbonate values using the conversion equation by Coplen (*115*). Then, the obtained SMOW δ^18^O carbonate values were converted into SMOW δ^18^O phosphate values using the Chenery *et al.* (*116*) equation. Eventually, the latter was converted into drinking water δ18O_DW_ values using the equation 6 by Daux *et al.* (*117*).

Converted δ^18^O_DW_ values were compared to the ^87^Sr/^86^Sr of the respective enamel sample and to the expected local/regional isotopic ranges within the investigated area. On the basis of the Online Isotopes in Precipitation Calculator (OIPC) (*58*) and on a previous bioarchaeological study (*54*), δ^18^O values are overall spanning from −7.0 to −11.4‰ throughout the research area. More elevated values may indicate a nonlocal origin. Nonetheless, we cannot exclude that other factors such as a disease, the breastfeeding effect in the case of early mineralizing teeth, or the brewing, boiling, or stewing effects, affected the isotopic composition of teeth showing high δ^18^O values (*36*). MIL1_187 is the only outlier in this case (δ^18^O_DW_, −6.2‰). The second molar that was analyzed was likely formed after a potential breastfeeding period, while the ^87^Sr/^86^Sr and δ^13^C values obtained from the same enamel sample are reliable. It is therefore unlikely that this outlier value was caused by alteration. It seems equally unlikely that this would be the only individual in the region to be affected by the brewing, boiling, or stewing effects. A nonlocal origin is thus considered the most likely interpretation for this individual.

### Strontium isotope baselines at each site

Enamel ^87^Sr/^86^Sr values were compared site by site with the Sr isotopic range considered as respective local baselines. Because of a general lack of adequate samples for the determination of bioavailable strontium as defined in recent reviews (*34*, *97*), the site-specific ranges for ^87^Sr/^86^Sr values as described below site by site are estimations based on published environmental and bioarchaeological isotope data from the region and on their relationship to the local geologies. These estimations may be improved in future research with a systematic sampling strategy including more modern plants and archaeological material. These samples were not available for this study, and the presented ranges are therefore the most reliable data available to date.

At Bocheniec, the geological substrate consists of Upper Jurassic limestones characterized by very unradiogenic strontium isotopic compositions, with values ranging from 0.7068 to 0.7073. These limestones are covered by a very thin layer of Pleistocene sands. Water from the Wierna River was sampled 4 km downstream from Bocheniec and yielded a strontium isotope ratio of 0.709197. Therefore, plausible human signatures should fall between 0.7073 and 0.7092, consistent with the signature of individual MIL1_177, which was interpreted as the only local individual at this site. Human signatures ranging from 0.7104 to 0.7116 thus fall outside the baseline range.

At Dacharzów 1, the geological basement consists of Middle Cambrian and Lower Ordovician rocks. The signatures of these rocks are known and were published by Walczak and Belka (*118*). The Cambrian rocks are overlain by Quaternary sediments, mainly loess, which have a highly radiogenic composition with ^87^Sr/^86^Sr signatures above 0.72 throughout the region. Locally along the Opatówka River valley, where Dacharzów is located, thin layers of Miocene quartz sands occur between loess and the Cambrian. Their composition was measured near Świniary and ranged between 0.7140 and 0.7150. In summary, the entire geological bedrock at Dacharzów has a highly radiogenic composition, with signatures above 0.7140. The waters of the Opatówka River measured in Wąworków yielded 0.7119. Accounting for the effect of modern artificial fertilizers, local surface water likely had values around 0.7122 to 0.7123 in historical times. Except from the interpolated and hence not verified estimations from the global isoscape model by Bataille *et al.* (*59*), there is no other source of strontium in the entire environment with signatures lower than 0.7122. Individuals with ^87^Sr/^86^Sr values lower than 0.7122 are thus outside the baseline range. This is supported by human signatures from the village of Sadowie (*119*), located near the Opatówka River, which shares the same geological bedrock as Dacharzów. The signatures of local people and animals there have values between 0.7124 and 0.7138 (*119*). The baseline range at Dacharzów 1 nevertheless deserves confirmation from further baseline samples.

At Pielgrzymowice, the geological substrate consists of Upper Cretaceous marly limestones and marls (with unradiogenic signatures around 0.7076 to 0.7080) covered by loess deposits with highly radiogenic isotopic compositions (*118*). Considering that the groundwater aquifers are situated within the Upper Cretaceous marls, it can be assumed that the local surface waters have a composition similar to that of the Szreniawa River [i.e., around 0.7086 (*81*, *120*)], which drains an area of very similar geology located directly east of Pielgrzymowice. The ^87^Sr/^86^Sr values of local individuals thus probably span from 0.7090 and 0.7105. At Słonowice, a slightly larger range from 0.7086 to 0.7112 can be expected.

At Miechów, the geological substrate consists of Upper Cretaceous sediments covered by up 20-m-thick layers of Pleistocene loess. The Upper Cretaceous sediments have a unradiogenic composition with signatures between 0.7073 and 0.7076, while loess has high signatures, exceeding 0.7200 (*118*). Therefore, the geological substrate contains sediments with extremely different isotopic compositions, which does not mean that the input of Sr from both sediment types to the environment is similar. The water of the Szreniawa River, measured downstream of Miechów, had, for example, an ^87^Sr/^86^Sr ratio of 0.7086 (*81*, *120*). In this context, limestones have a greater Sr input into the environment than loess; hence, the extent of mixing during the generation of human signatures is considered to be closer to the signatures of water and of the dominant Upper Cretaceous sediments. Individuals with ^87^Sr/^86^Sr signatures around 0.7080 to 0.7102 therefore represent local individuals. Because of the same geological and environmental settings at Pieczeniegi/Rzemięcice, this site exhibits the same Sr isotope baseline range as at Miechów.

At Żerniki Górne, the geological substrate consists of Maastrichtian (Uppermost Cretaceous) carbonates and marls characterized by Sr isotopic signatures of 0.7077 to 0.7078 (*118*), without loess deposits with highly radiogenic isotopic compositions. This explains why the signatures of local individuals are expected to be lower than those observed at Miechów, Pielgrzymowice, or Słonowice, i.e., between 0.7080 and 0.7100 (*16*, *121*).

The sites and individuals from Kraków Nowa Huta-Cło (and Kraków Nowa Huta-Mogiła) are influenced by the very complex geology around Kraków. The geological substrate predominantly consists of Upper Jurassic carbonates and various marine Miocene deposits, all of which are characterized by ^87^Sr/^86^Sr signatures below 0.7090. The Upper Jurassic limestones, which are responsible for the town’s most prominent morphological features, exhibit very low ^87^Sr/^86^Sr values of 0.7068 to 0.7069 (*118*). On the uplands surrounding the city, the Jurassic and Miocene deposits are covered by loess layers with a highly radiogenic composition. Riverine water from the Vistula River, measured slightly upstream of the town, yielded a value of 0.709655 ± 9 (*81*). This value certainly does not reflect the original isotopic composition because saline waters from coal mines in Silesia are discharged into the Vistula ∼60 km upstream of Kraków (*81*). These waters are characterized by ^87^Sr/^86^Sr values above 0.7105. However, in historical times, the Vistula had presumably a less radiogenic composition. We can therefore estimate a local ^87^Sr/^86^Sr range around 0.7080 to 0.7100 for Kraków Nowa Huta-Cło.

At Świniary Stare, the geological substrate consists of Lower Cambrian shales and sandstones overlain by layers of Miocene carbonate sands and Pleistocene sands. The Sr isotopic composition of the Lower Cambrian rocks, measured at numerous localities in the region, is characterized by very high radiogenic signatures, ranging from 0.7200 to 0.7700 (*80*, *81*, *122*). In contrast, the Miocene carbonates are relatively unradiogenic, with ^87^Sr/^86^Sr values around 0.7088 to 0.7090 (*118*). The Pleistocene sands again exhibit a highly radiogenic composition. The numerous water sources affecting the environment at and around Świniary Stare have not yet been measured. Overall, the predominance of radiogenic sediments explains why local individuals from Świniary Stare are characterized by relatively high signatures and the narrowest possible range for local ^87^Sr/^86^Sr values possibly would be between 0.7106 and 0.7113.

The sites at Zubowice and Brodzica share the same geological substrate, consisting of Uppermost Cretaceous limestones with ^87^Sr/^86^Sr signatures of 0.7078 (*118*), locally covered by layers of loess. Considering the strontium reservoirs present in this region, it can be argued that surface waters possibly have Sr isotopic signatures higher than those of the geological substrate (which hosts the aquifers) but lower than those of precipitation waters, producing a plausible local range between 0.7078 and 0.7092. On the basis of published references, the local ^87^Sr/^86^Sr spectrum for the site at Koszyce can be estimated at around 0.7087 to 0.7111 (*120*). The interpretation of mobility patterns at Gustorzyn derives from Pospieszny *et al.* (*54*).

### Radiocarbon analyses on collagen

The same bone collagen produced in the framework of stable isotope analyses was used for the radiocarbon analyses of individuals MIL1_205, MIL1_207, and MIL1_233. The measurements were performed at the Vilnius Radiocarbon Laboratory of the FTMC at Vilnius. The graphitization procedure of the samples was performed using an Automated Graphitization Equipment AGE-3 (IonPlus AG). A low-energy accelerator mass spectrometer from IonPlus AG (Switzerland) was used for radiocarbon measurements. The accuracy of the measured ^14^C/^12^C ratio was better than 0.3%. Reference materials were NIST-OXII and phthalic anhydride.

All previously published and newly produced radiocarbon dates (before the present) were homogeneously (re)calibrated in January 2026 using OxCal v.4.4.4 (*123*), r.5 and the IntCal20 calibration curve based on atmospheric data from Reimer *et al.* (*124*).

The date FTMC-XY56-6 (3097 ± 31 B.P.) replicates Poz-104939 (3200 ± 34 B.P.) (*16*). The around hundred-year difference between the two calibrated dates, 1356 ± 45 cal BCE and 1473 ± 37 cal BCE, falls within measurement errors and variability observed elsewhere between dates from the same bones and, as in this context, from different bones of the same individual (*125*). To avoid confusion between this paper and previous studies on the same individuals, the previously published date was kept as reference for this individual (i.e., Poz-104939) (*16*).

The OxCal function for KDE models (KDE_Model) (*60*) was used to more accurately determine the timing of burial activities and related mobility patterns at the various sites and at the regional level. When determining the earliest date for millet consumption at each site, the date used at Słonowice derives from the radiocarbon-dated individual clearly identified as millet consumer (MIL1_327) because the individual who changed diet over life (MIL1_331) is not radiocarbon dated and has a bone collagen δ^13^C value still below −18.0‰ (Fig. 2).

### Paleoproteomics

All chemicals used for paleoproteomics were obtained from Sigma-Aldrich (St. Louis, MO, USA). The tooth surface was clean with molecular water via wiping. Then, the tooth crown was submerged in the cap of a 1.5-ml Eppendorf tube containing 120 μl of 10% trifluoroacetic acid (TFA) (v/v) during 1 min for the preetching. The same operation was reproduced for 15 min using a new 1.5-ml Eppendorf tube cap containing fresh 10% TFA (v/v) solution (120 μl) for the main etching. Only the main etching peptide solution was collected for the analysis. Peptides were purified using a C18 resin–loaded StageTips. StageTips were conditioned with 150 μl of pure methanol, spun to pass at 13,300 rpm with 150 μl of 0.1% TFA in acetonitrile (ACN)/0.1% TFA in molecular water (80/20, v/v), and spun to pass at 13,300 rpm. Peptide solutions were loaded on the StageTips before spun to pass at 13,300 rpm. StageTips were rinsed with twice 150 μl of 0.1% TFA (v/v) and spun to pass. Peptides were lastly eluted using 40% ACN in 0.1% TFA (v/v) and freeze dried at −80°C.

The tooth was clean in Milli-Q ultrapure water via sonication. A handheld clean drill was used to extract 20 to 50 mg of enamel. Enamels were demineralized overnight using 0.5 ml of 10% TFA (v/v) in a 1.5-ml Eppendorf tube. This 24-hour demineralization step was repeated to ensure the highly biomineralized remains were adequately demineralized. After demineralization, supernatants were spun for 30 min at 13,300 rpm. Peptides were purified using a C18 resin–loaded ZipTips. ZipTips were conditioned three times using 50 μl of pure ACN and then three times using 50 μl of 0.1% TFA (v/v). Peptide solutions were loaded 10 times on the ZipTips. ZipTips were rinsed six times with 50 μl of 0.1% TFA (v/v) before being lastly eluted using 50 μl of 40% ACN in 0.1% TFA (v/v) and freeze dried at −80°C.

The protein extraction mode used for each sample is detailed on the results file located on the Supplementary Materials (data S3). All protein extraction was performed in a paleoproteomic clean laboratory. Extraction blanks were processed alongside each sample set.

Before the liquid chromatography–high-resolution MS (LC-HRMS) paleoproteomic analysis, peptides were reconstituted in 10 μl of 4% ACN containing 1% formic acid (FA) (v/v).

An UltiMate 3000 RSLCnano LC system (Thermo Fisher Scientific) equipped with a trapping cartridge (μ-Precolumn C18 PepMap 100, 300 μm internal diameter × 5 mm, 5-μm particle size, 100-Å pore size; Thermo Fisher Scientific) and an analytical column (nanoEase M/Z HSS T3, 75 μm i.d. × 250 mm, 1.8-μm particle size, 100-Å pore size; Waters). Samples were trapped at a constant flow rate of 30 μl/min using 0.05% TFA in Milli-Q ultrapure water for 6 min. After switching in line with the analytical column, which was preequilibrated with solvent A [3% dimethyl sulfoxide (DMSO) and 0.1% FA in Milli-Q ultrapure water (v/v)], the peptides were eluted at a constant flow rate of 0.3 μl/min using a 90-min gradient of increasing solvent B concentration [3% DMSO and 0.1% FA in ACN (v/v)].

The outlet of the analytical column was directly coupled to an Orbitrap Exploris 480 mass spectrometer (Thermo Fisher Scientific) equipped with a Nanospray Flex ion source, operating in positive ion mode. Peptides were introduced into the instrument using a Pico-Tip emitter (360 μm outer diameter × 20 μm inner diameter; 10-μm tip; CoAnn Technologies) with a spray voltage of 2.5 kV. The capillary temperature was maintained at 275°C. Full MS (MS1) scans were acquired in profile mode over a mass/charge ratio (*m/z*) range of 350 to 1400, with a resolution of 120,000 at *m/z* 200 in the Orbitrap. The acquisition was performed on data-dependent top 10 mode. The automatic gain control (AGC) target was set to “custom” with a normalized AGC target of 300% and a maximum injection time of 25 ms. MS/MS scans were acquired in profile mode with a resolution of 60,000. The AGC target was set to “custom,” with a normalized value of 200%. The maximum injection time was 118 ms. A normalized higher-energy collisional dissociation energy of 30% was applied for fragmentation. Dynamic exclusion was set to 20 s.

The analysis of MIL1_180 failed because of a technical issue with the mass spectrometer. All raw files generated by LC-HRMS were processed using MaxQuant (*126*) (version 2.7.0.0 or higher). The peptide identification was performed setting the digestion parameter to unspecific. No fixed posttranslational modifications were set. Deamidation (NQ) and oxidation (MP) were included as possible variable modifications. The minimum length for unspecific peptides search was set to six amino acids. The minimum score for unmodified peptides was set to 40 to filter out peptide spectrum matches with a low-quality score. The FASTA file was made with *Homo sapiens* AMELX (Q99217) and AMELY (Q99218) proteins.

The biological sex identification was performed using the untargeted paleoproteomic sex identification R (version 4.5.1) code with a Shiny user interface [https://github.com/MarineMorvan/SexPeptID; see also (*127*)]. Sex-specific peptides from AMELX and AMELY proteins were selected on the basis of molecular sexual dimorphism, with the presence of methionine-45 (AMELY) or its absence (AMELX) (*128*–*130*). Both peptides.txt and msms.txt output files were uploaded from MaxQuant without requiring any preprocessing or modification.

### Statistical analysis

Because we are interested in the analysis of variance between groups and one-way analysis of variance (ANOVA) tests are suitable for the comparison of two groups or more, these tests were performed using the R software (version 4.5.1) to test the significance of isotopic differences between selected categories, e.g., C_3_ diet versus C_4_ inputs, individuals with enamel ^87^Sr/^86^Sr or δ^18^O values falling “inside the range” or “outside the range” determined for each site, and “adult males” versus “adult females.” The detailed results are listed in Table 2, and the data used are derived from data S1.

### Information about the map background

To generate the maps on Fig. 1 using QGIS, a digital elevation model (DEM) was used as follow: a resampled 500-m DEM from the Global Multi-Resolution Terrain Elevation Data 2010 [GMTED2010 (*131*), last accessed 29 May 2026]. Rivers and lakes layers are made with Natural Earth, using free vector and raster map data at naturalearthdata.com, last accessed 29 May 2026.
