# Supplementary material for: Multiproxy bioarchaeological analyses reveal subsistence shift in Bronze Age Central Europe
Source: Sci Adv. 2026 Sep 9;12(37):eaeh1079. doi: 10.1126/sciadv.aeh1079 (PMC13557095; doi:10.1126/sciadv.aeh1079)
Supplement: Supplementary file 1 [file sciadv.aeh1079_sm.pdf]

Science Advances
AAAS

Supplementary Materials for

Multiproxy bioarchaeological analyses reveal subsistence shift in Bronze Age Central Europe

Margaux L. C. Depaermentier *et al.*

Corresponding author: Margaux L. C. Depaermentier, margaux.depaermentier@if.vu.lt;
Giedrė Motuzaitė Matuzevičiūtė, giedre.motuzaite@gmail.com

Sci. Adv. **12**, eaeh1079 (2026)
DOI: 10.1126/sciadv.aeh1079

The PDF file includes:

Supplementary Text S1
Figs. S1 to S8
Legends for data S1 to S3

Other Supplementary Material for this manuscript includes the following:

Data S1 to S3

Supplementary Text S1.

Provenance of the archaeological material.

The archaeological skeletal material used in this study for multiple stable and radiocarbon analyses originates from various storage institutions (see also Data S1), with which agreements were signed to access, collect, sample and analyse the material. The agreements also include the permission to publish the resulting data and knowledge, and all person involved in this material acquisition are integrated as co-author in the paper.

The archaeological skeletal material from Bocheniec 2, Pieczeniegi/Rzemiędzice and Świniary Stare 1 are stored and can be freely accessed at the Archaeological Museum in Krakow. Samples were validated for authenticity and aged/dated by: A. Szczepanek, A. Matoga, J. Górski, P. Makarowicz and P. Jarosz. Samples from Bocheniec 2 were collected by A. Szczepanek in 2016, samples from Pieczeniegi/Rzemiędzice, Świniary Stare 1 were collected by A. Juras, M. Chyleński in 2016.

The skeletal material from Pielgrzymowice 9, Słonowice 5, Żerniki Górne 1 originates from the Institute of Archaeology and Ethnology of the Polish Academy of Science in Cracow and can be freely accessed from this Institution. Samples were validated for authenticity and aged/dated by: A. Szczepanek, P. Włodarczak, K. Tunia, A. Lasota, J. Wilczyński. Samples from Pielgrzymowice 9, Żerniki Górne 1 were collected by A. Szczepanek, A. Juras, M. Chyleński in 2016, samplaes from Słonowice 5 were collected by A. Szczepanek in 2024.

The material from Dacharzów 1, Podlodów 2 and Zubowice 1A is stored at the Institute of Archaeology from the Maria Curie-Skłodowska University in Lublin and can be freely accessed from this Institution. Samples were validated for authenticity and aged/dated by: H. Taras, P. Makarowicz, A. Szczepanek. Samples from Dacharzów 1, Podlodów 2 were collected by H. Taras, A. Juras, M. Chyleński in 2016, samples from Zubowice 1A were collected by A. Szczepanek in 2016.

The skeletons from Koszyce 3, Kraków Nowa Huta Cło 65 and Kraków Nowa Huta-Mogiła 55 originate from the Nowa Huta Branch of the Archaeological Museum in Cracow (Branice) and can be freely accessed from this Institution. Samples were validated for authenticity and aged/dated by: I. Wójcik, J. Górski, P. Makarowicz. Samples from Koszyce 3 were collected by I. Wójcik in 2016 and 2024, samples from Kraków Nowa Huta Cło 65 were collected by A. Juras, M. Chyleński in 2016, samples from Kraków Nowa Huta-Mogiła 55 were collected by A. Szczepanek in 2016 and 2024.

The material from Miechów 3 originates from the Jagiellonian University and can be freely accessed from this Institution. Samples were validated for authenticity and aged/dated by M. Nowak and collected by him in 2021.

The material from Gustorzyn 1 originates from the Museum of Archaeology and Ethnography in Łódź and can be freely accessed from this Institution. Samples were validated for authenticity

and aged/dated by: R. Grygiel and A. Szczepanek. Samples were collected by A. Szczepanek, A. Juras, M. Chyleński in 2016

The material from Brodzica 19 originated from the Stanisław Staszic Museum in Hrubieszów. Samples were validated for authenticity and aged/dated by: H. Taras and sampled by A. Juras, M. Chyleński in 2016.

The Museum in Lublin furthermore provided the samples of two skeletons from Kazimierzów 3, which were then excluded from the study because we could not extract any collagen from those samples despite several attempts.

All samples were shared with the Bioarcheology Research Center at Vilnius University in 2024, in the framework of the European Research Council Consolidator Grant 101087964 (awarded by GMM). The leftover of the material sent for analyses is then returned to the original storage institution.

Supplementary Figures

Fig. S1.

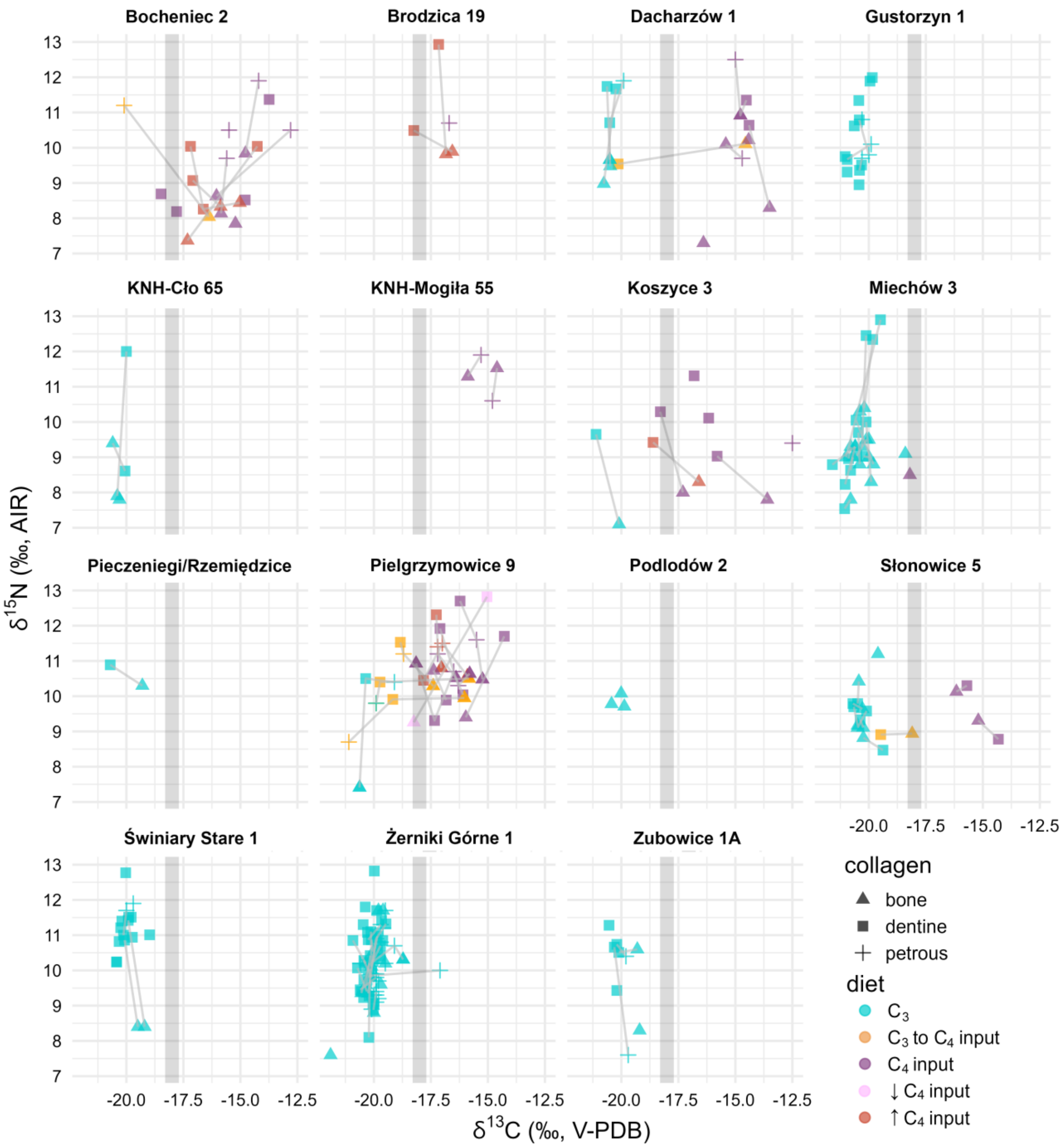

Multi-tissue collagen δ^{13}C and δ^{15}N values reveal dietary shifts at the individual level. The samples from the same individual are connected with a grey line. The shape of the signature informs about the tissue in which the collagen isotopic composition was measured. The grey horizontal shaded areas mark the δ^{13}C value at around -18.0‰, a common threshold for identifying C_4 dietary inputs in Poland (see methods section). The signature color reflects the resulting dietary information for each individual, which was refined using associated enamel δ^{13}C values (see Fig. S2 and methods section). Individual raw data and their interpretation in terms of diet are listed in Data S1.

Fig. S2.

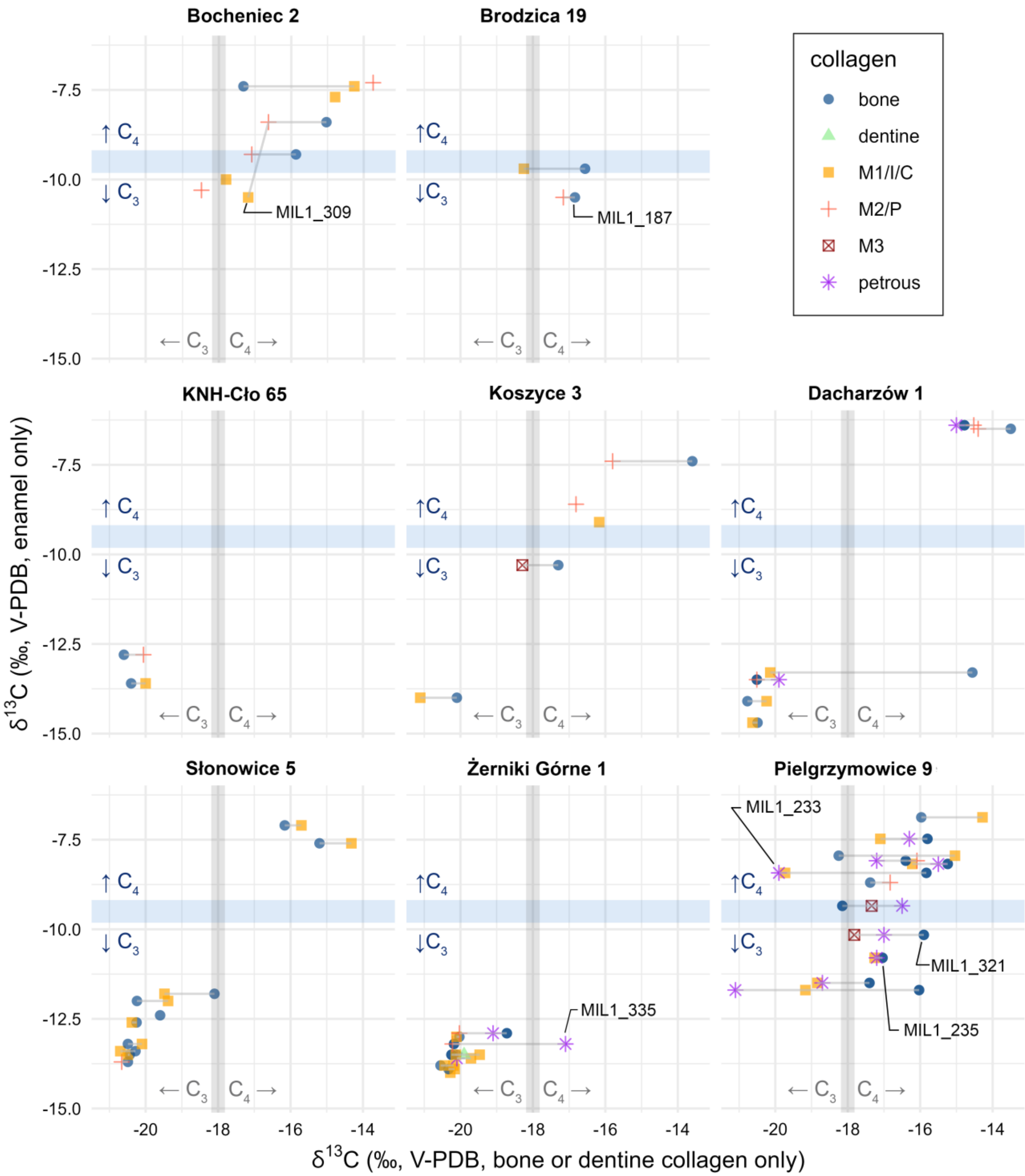

Dental enamel carbonate δ^{13}C values related to the collagen δ^{13}C values of all other samples from the same individual for each site and each individual dated to after 1700 BCE. The color and shape of the points inform about the body part from which the collagen δ^{13}C value derives. The samples belonging to the same individual are connected with a grey line. Bone data (including petrous) are informing only about collagen δ^{13}C values on the x axis, while they plot at the same place of their associated enamel δ^{13}C values on the y axis. This is to

highlight dietary changes over life. The thresholds for C_4 diet identification are depicted in blue shade for enamel carbonate $\delta^{13}C$ values and by the grey shade for collagen $\delta^{13}C$ values. The multi-tissue approach improves the interpretation of ambiguous values with respect to these thresholds (see individual labels and related explanations in the methods section).

Fig. S3.

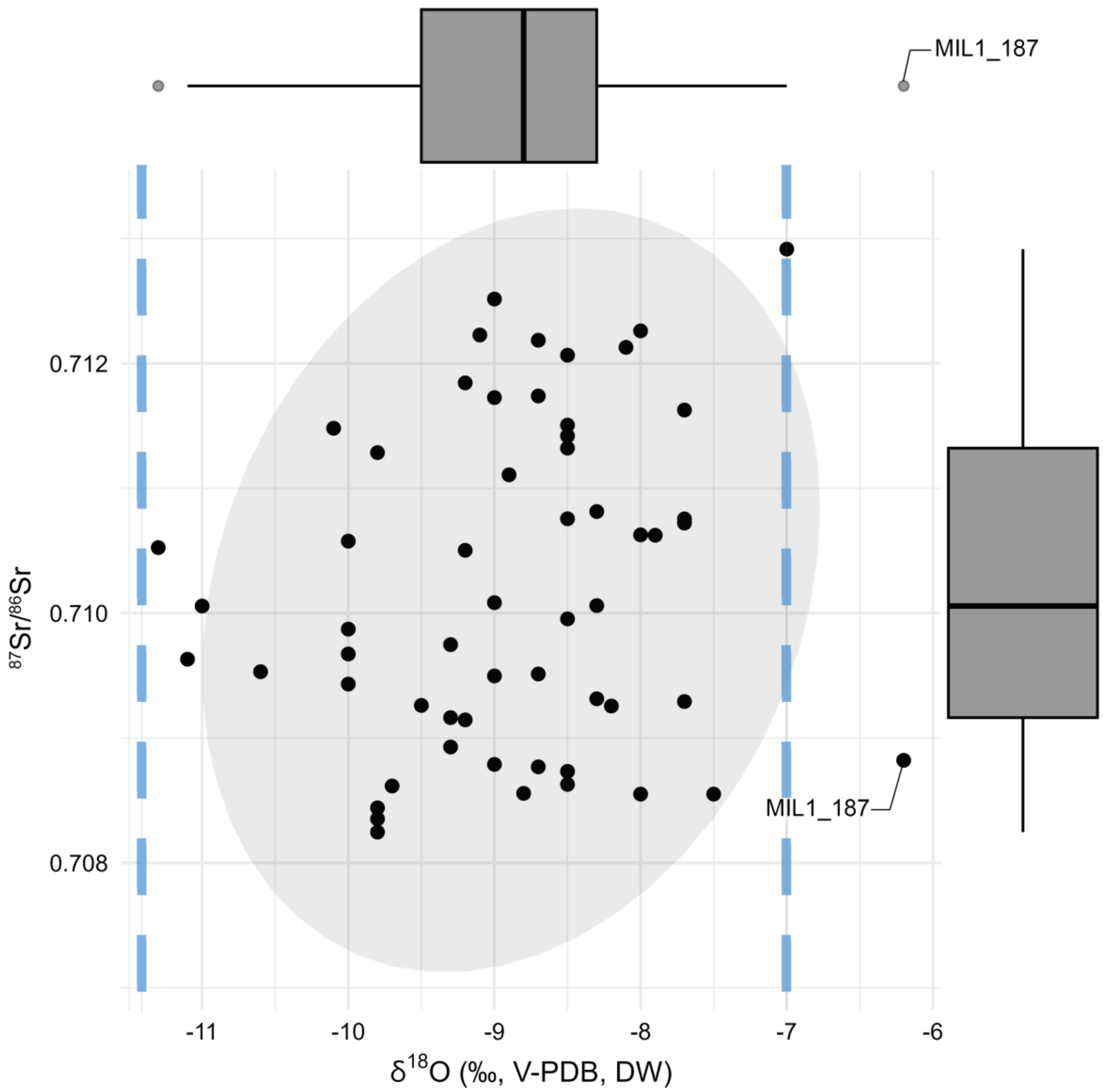

Dental enamel $\delta^{18}O$ and $^{87}Sr/^{86}Sr$ values for each individual. The individual's $^{87}Sr/^{86}Sr$ values mostly result from a mean of two measurements (see Data S1 and the methods section). The ellipse shows a confidence level of 95%. Individuals with low $\delta^{18}O_{DW}$ values still fit within the range expected for the region. The only proper outlier in $\delta^{18}O_{DW}$ is labelled and its identification as outlier is described in the methods section. The boxplots at the edges exemplify the various distribution of $\delta^{18}O_{DW}$ (top panel) and $^{87}Sr/^{86}Sr$ values (right panel) within the whole region. The blue dashed lines represent the limits of the regional oxygen isotopic range expected from modern precipitation (defined in the methods section). The $^{87}Sr/^{86}Sr$ baselines are defined in the methods section and vary between sites (see also Fig. S4).

Fig. S4.

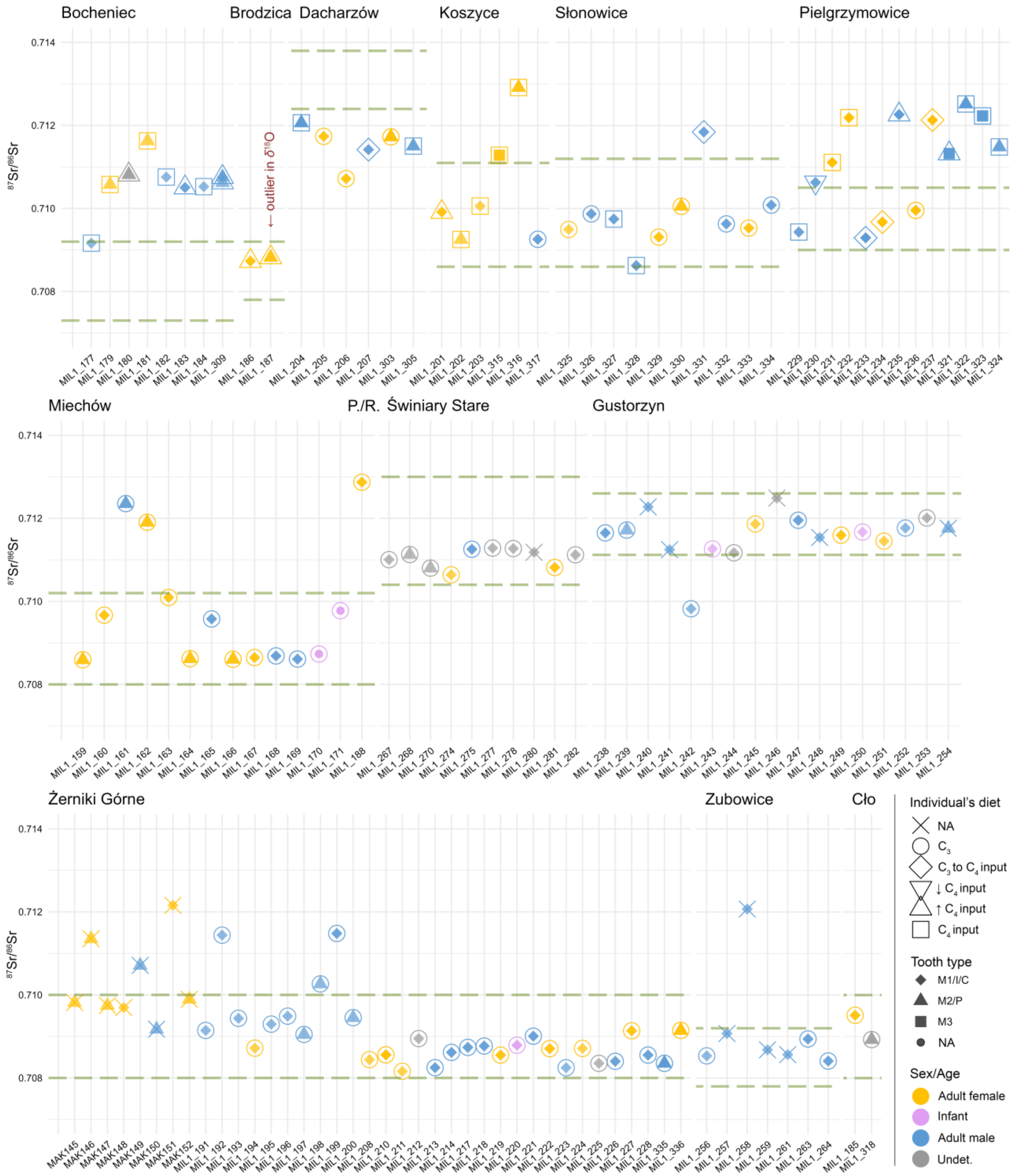

Dental enamel ^{87}Sr/^{86}Sr values of each individual compared to the local strontium isotopic baseline at each site and associated with information on sex, age and diet. The individual's ^{87}Sr/^{86}Sr values mostly result from a mean of two measurements (see Data S1 and the methods section). The green dashed lines represent the estimated upper and lower limits of each site's local ^{87}Sr/^{86}Sr range as defined in the methods section: these are no sharp cut-off values but

rather serve as orientation. The color of the dots informs on the age and sex of the individuals. The closed signatures inform on the tooth type in which the $^{87}Sr/^{86}Sr$ was measured (tooth: undetermined; M1/I/C: permanent first molar or incisive or canine; M2/P: permanent second molar or premolar; M3: permanent third molar). The open signatures around the closed ones inform on the individual's diet as defined from the multi-tissue stable C and N isotope data. Sites with isotopic evidence for millet consumption are grouped in the top panel. The middle and lower panels reflect sites without clear isotopic evidence for C_4 intake.

Fig. S5.

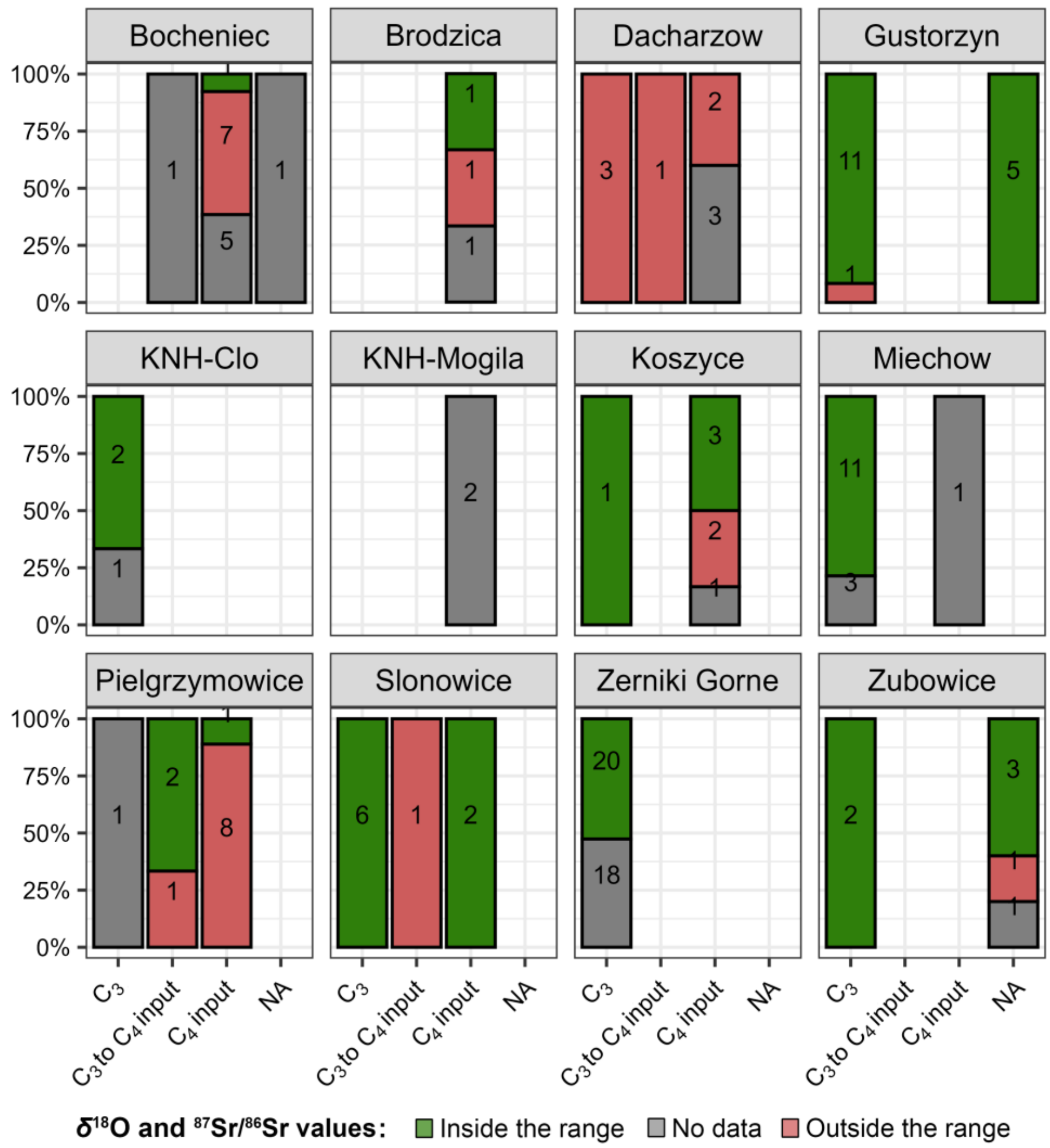

Diversity of Sr isotopic backgrounds at each site depending on dietary categories for individuals dated to after 1700/1600 BCE. The counts and proportions of individuals with various Sr isotopic backgrounds and dietary habits reveals a great diversity of scenarios and combinations at the site level.

Fig. S6.

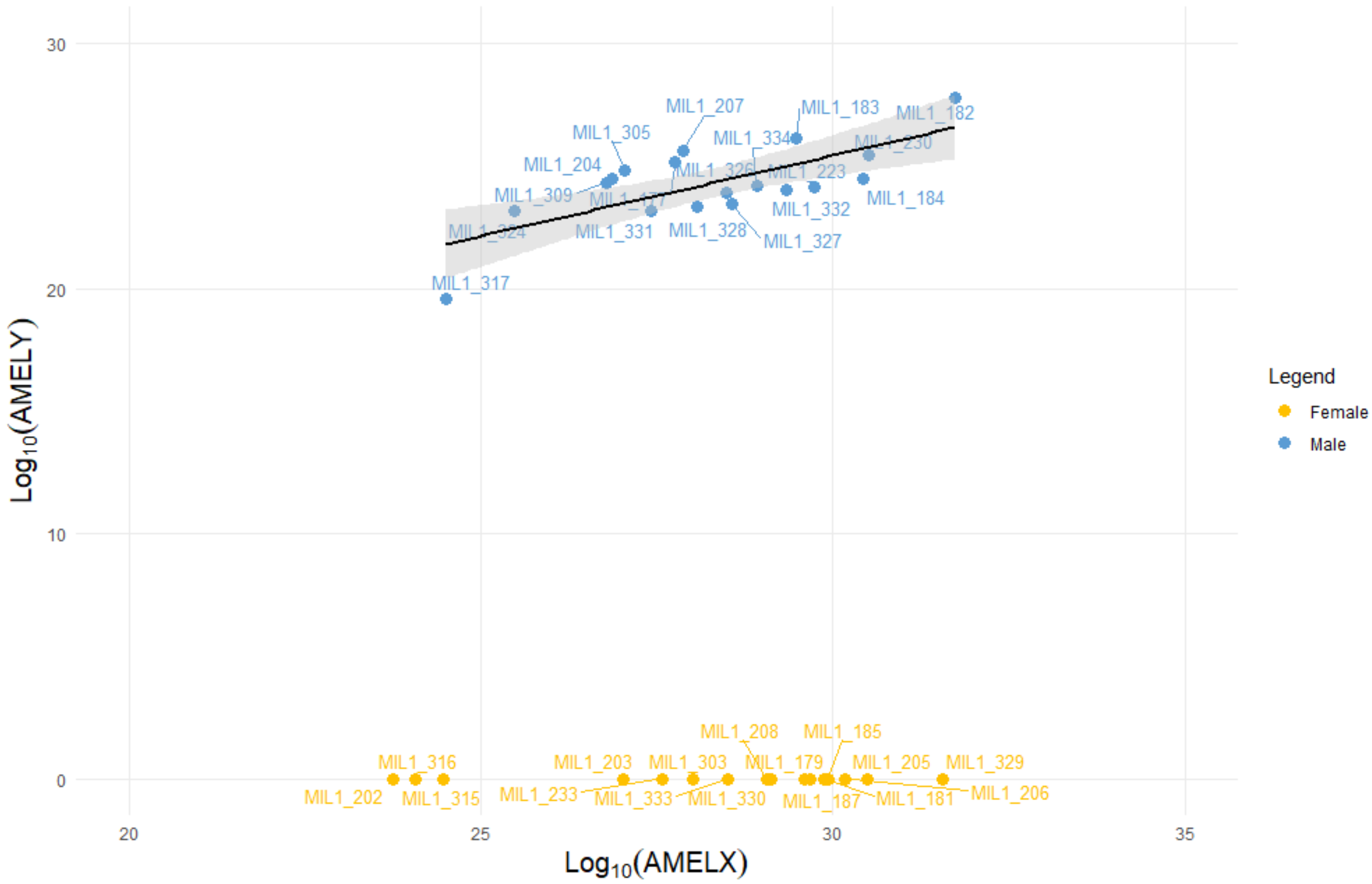

The AMELX and AMELY intensities (Log_{10}) of selected sex-dependent peptides focused on the presence or absence of methionine-45. The solid line shows the linear regression of log_{10}(AMELX) versus log_{10}(AMELY) for males, and the shaded area represents the 95% confidence interval of the estimated mean. Intensities are reported in the supplementary material (Data S3).

Fig. S7.

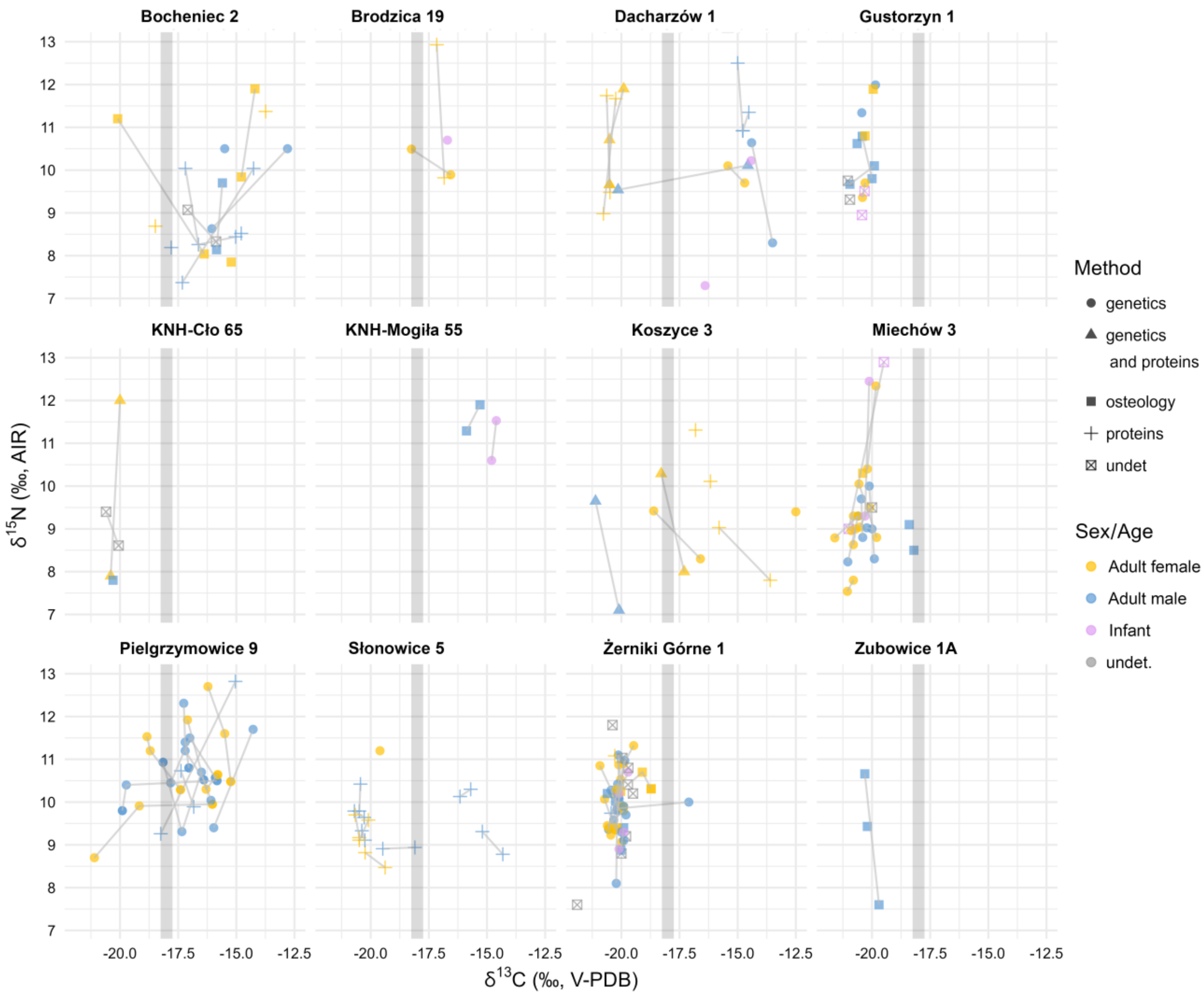

Collagen δ^{13}C and δ^{15}N values of adult males, adult females, infants, and unsexed individuals dated from after 1700 BCE at the various sites. The samples belonging to the same individual are connected with a light grey line. The tissue type of each sample can be found in Data S1 and Fig. S1. The color of the dots reflects the age and sex categories of the individuals. The shape of the dots represents the method used for sex identification. The grey vertical shaded areas mark the δ^{13}C value at around -18.0‰, a common threshold for identifying C_4 dietary inputs in Poland (see methods section).

Fig. S8.

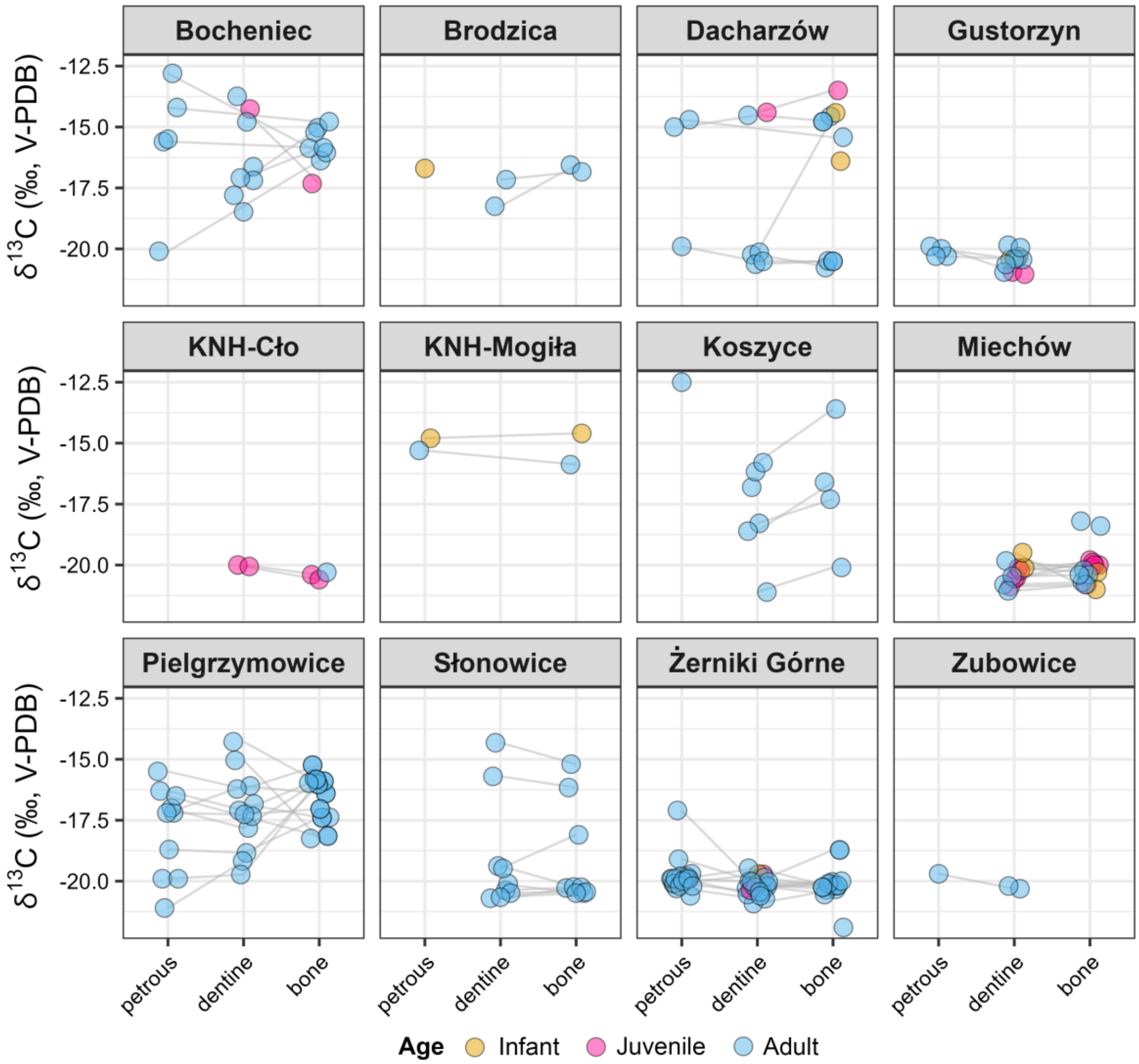

Collagen $\delta^{13}C$ values of infant, juvenile and adult individuals dated from after 1700 BCE at the various sites. The samples belonging to the same individual are connected with a light grey line. The petrous bone data represent the in-utero life, the dentine data represent various stages of the childhood and the bone samples represent the last years of life – which still represents childhood for the infant individuals. Details can be found in Data S1.

Data S1. (separate file)

Complete dataset including both newly generated and published data. The references abbreviated in this dataset are available from the reference list of the main manuscript. The file lists all information for each individual, including archaeological, anthropological, and genetic background data. It shows the raw data for all newly generated and published isotope, paleoproteomic, and radiocarbon data and provides the related interpretation. The storage institution of the archaeological skeletal material is specified.

Data S2. (separate file)

Results of the KDE models. (A) KDE model for all C_4-consumers. **(B)** KDE model for all $\delta^{18}O$ and $^{87}Sr/^{86}Sr$ outliers at sites with carbon isotopic evidence for C_4-diet. **(C)** KDE model for Dacharzów. **(D)** KDE models for Pielgrzymowice. **(E)** KDE models for Koszyce. **(F)** KDE model for Bocheniec. **(G)** KDE model for Brodzica. **(H)** KDE model for Słonowice.

Data S3. (separate file)

Paleoproteomics data. Summed intensities of sex-specific peptides used for paleoproteomics sex identification.
